# Structure and Fc-Effector Function of Rhesusized Variants of Human Anti-HIV-1 IgG1s

**DOI:** 10.3389/fimmu.2021.787603

**Published:** 2022-01-06

**Authors:** William D. Tolbert, Dung N. Nguyen, Marina Tuyishime, Andrew R. Crowley, Yaozong Chen, Shalini Jha, Derrick Goodman, Valerie Bekker, Sarah V. Mudrak, Anthony L. DeVico, George K. Lewis, James F. Theis, Abraham Pinter, M. Anthony Moody, David Easterhoff, Kevin Wiehe, Justin Pollara, Kevin O. Saunders, Georgia D. Tomaras, Margaret Ackerman, Guido Ferrari, Marzena Pazgier

**Affiliations:** ^1^ Infectious Disease Division, Department of Medicine of Uniformed Services University of the Health Sciences, Bethesda, MD, United States; ^2^ Department of Surgery, Duke University School of Medicine, Durham, NC, United States; ^3^ Human Vaccine Institute, Duke University School of Medicine, Durham, NC, United States; ^4^ Center for Human Systems Immunology, Duke University School of Medicine, Durham, NC, United States; ^5^ Thayer School of Engineering, Dartmouth College, Hanover, NH, United States; ^6^ Division of Vaccine Research, Institute of Human Virology, University of Maryland School of Medicine, Baltimore, MD, United States; ^7^ Public Health Research Institute, New Jersey Medical School, Rutgers University, Newark, NJ, United States; ^8^ Department of Pediatrics, Duke University School of Medicine, Durham, NC, United States; ^9^ Department of Medicine, Duke University School of Medicine, Durham, NC, United States

**Keywords:** antibody engineering, rhesusization, Fc-effector function, non-human primates, HIV

## Abstract

Passive transfer of monoclonal antibodies (mAbs) of human origin into Non-Human Primates (NHPs), especially those which function predominantly by a Fc-effector mechanism, requires an *a priori* preparation step, in which the human mAb is reengineered to an equivalent NHP IgG subclass. This can be achieved by changing both the Fc and Fab sequence while simultaneously maintaining the epitope specificity of the parent antibody. This Ab reengineering process, referred to as rhesusization, can be challenging because the simple grafting of the complementarity determining regions (CDRs) into an NHP IgG subclass may impact the functionality of the mAb. Here we describe the successful rhesusization of a set of human mAbs targeting HIV-1 envelope (Env) epitopes involved in potent Fc-effector function against the virus. This set includes a mAb targeting a linear gp120 V1V2 epitope isolated from a RV144 vaccinee, a gp120 conformational epitope within the Cluster A region isolated from a RV305 vaccinated individual, and a linear gp41 epitope within the immunodominant Cys-loop region commonly targeted by most HIV-1 infected individuals. Structural analyses confirm that the rhesusized variants bind their respective Env antigens with almost identical specificity preserving epitope footprints and most antigen-Fab atomic contacts with constant regions folded as in control RM IgG1s. In addition, functional analyses confirm preservation of the Fc effector function of the rhesusized mAbs including the ability to mediate Antibody Dependent Cell-mediated Cytotoxicity (ADCC) and antibody dependent cellular phagocytosis by monocytes (ADCP) and neutrophils (ADNP) with potencies comparable to native macaque antibodies of similar specificity. While the antibodies chosen here are relevant for the examination of the correlates of protection in HIV-1 vaccine trials, the methods used are generally applicable to antibodies for other purposes.

## Introduction

The only vaccine to show efficacy although limited against HIV-1 infection has been the RV144 vaccine trial in Thailand consisting of a canarypox ALVAC-HIV DNA prime and AIDSVAX B/E protein boost which showed an overall efficacy of 31.2% against HIV-1 acquisition (1). Secondary analyses of correlates of reduced risk of infection revealed the role played by antibody-dependent cellular-cytotoxicity (ADCC) and Fc effector functions against HIV-1 Env in the absence of high levels of Env-specific serum IgA (2–5). Breakthrough infections showed vaccine pressure on the V2 loop region of Env which could be seen by sequence changes in V2 (6–9). Many attempts to reproduce this protective effect with the same or similar vaccine regimens have succeeded in non-human primates (NHP) (10–18), but a study in humans did not show efficacy, potentially due to differences in adjuvant (MF59 vs. alum), and subtype and sequences of the immunogens (subtype C vs AE) (19, 20). While still under investigation, additional reasons for this discordance may include differences in the circulating strains in human populations versus those in NHP challenge stocks and most importantly, human-NHP interspecies differences in host genetics that may contribute to the type, magnitude, functionality and duration of the induced immune response. However, the HVTN 505 HIV-1 clinical efficacy trial consisting of a DNA and Adenovirus 5 vaccine regimen, despite lacking overall efficacy, did show a correlation between anti-Env serum IgG3, antibody dependent cellular phagocytosis (ADCP) and *in vitro* FcγRIIa engagement and a reduced risk of HIV-1 infection as well as a correlation between FcγRIIa engagement and a decreased viral load setpoint in breakthrough vaccinees (21), indicating that strategies to induce more potent antibody Fc effector functions in a greater proportion of vaccinees are needed.

Rhesus macaque (RM), *Macaca mulatta*, the most often used primate in NHP studies, shares approximately 93% genome identity with humans, but that high degree of similarity breaks down in immune system genes. There are significant differences in immunoglobulin (IgG) subclasses, Fc receptor diversity and Fcγ receptor (FcγR) expression patterns between human and RM that complicate the translation of experimental results in RM to humans (22–24). RM IgG subclasses are in general more similar to each other than are their human counterparts; both macaques and humans have four IgG subclasses, but macaques lack highly divergent subclasses such as human IgG3 with its long repetitive hinge region. Macaques also lack an FcγRIIIb gene which was the result of a gene duplication event that occurred after the divergence of macaques from other higher primates (25); humans have a FcγRIIIa and a FcγRIIIb receptor while macaques only have a FcγRIIIa receptor. Both have FcγRIIa (activating) and FcγRIIb (inhibitory) receptors. Although human IgGs can bind macaque FcγRs, these and other differences change the Fc/FcγR functional landscape between the two species.

Given the difficulty in reproducing RV144 vaccine limited efficacy results in other vaccine trials, it is important to be able to define mechanistic aspects of the correlates of protection conferred by the human vaccine in NHP. This is typically done for antibodies cloned from HIV-1 infected or vaccinated individuals by passive transfer of the monoclonal antibody (mAb) or mAb mix into NHP followed by a viral challenge to test its ability to protect against infection or to provide post-infection control. Many NHP passive transfer trials have been used to successfully test antibodies that function predominately by neutralization, where an Fc effector mismatch is less of an issue (26–28). In these studies, a mAb of human origin is directly transferred into NHP without reengineering to the equivalent RM IgG subclass. Although convenient, such approaches potentially suffer from anti-drug antibody (ADA) responses that can quickly remove species mismatched antibodies from circulation, thereby abrogating any functional effect. The longer the duration of the trial, the higher the probability of inducing such responses becomes (29). Furthermore, passive transfer testing of mAbs that function predominately by Fc-effector mechanisms, such as those from the RV144 trial, require both a minimization of the ADA response and a match of the Fc of the infused antibody to that of the host to more accurately mirror what is happening in macaque immunization trials. For human mAbs to be tested in RM this means rhesusization of both the Fc and Fab while simultaneously maintaining epitope specificity.

Here we describe a successful attempt to rhesusize a set of human mAbs targeting HIV-1 Env epitopes involved in potent Fc-effector mechanisms. This set includes a mAb targeting the linear gp120 V1V2 epitope isolated from a RV144 vaccinee (30), a gp120 conformational epitope within the Cluster A region isolated from a RV305 vaccinated individual, a RV144 vaccinee who had further boosts with RV144 immunogens in the RV305 vaccine trial that was designed to test if correlates of protection in RV144 could be boosted in RV144 vaccinees (31), and a linear gp41 epitope within the immunodominant Cys-loop region commonly targeted by most HIV-1 infected individuals (32). Antibodies binding to these three regions have been implicated in protection from infection from HIV-1, but not in neutralization of the virus. Structural analyses confirm that the rhesusized variants bind their respective Env antigens with almost identical specificity as their human counterparts—preserving epitope footprints and most antigen-Fab atomic contacts with constant regions folded as in control RM IgG1. Functional analyses confirm their Fc effector functionality, including the ability to mediate Antibody Dependent Cell-mediated Cytotoxicity (ADCC), Antibody Dependent Cellular Phagocytosis by monocytes (ADCP), and Antibody Dependent Cellular Phagocytosis by neutrophils (ADNP) with potencies comparable to native macaque antibodies of similar specificity. This will facilitate the testing of their impact on the virus in the future in an animal model. While the antibodies chosen here are relevant for examining the correlates of protection in HIV-1 trials the methods used are generally applicable to antibodies for other purposes.

## Material and Methods

### Rhesusization of Antibodies

Human variable regions were rhesusized as previously described with minor modifications (33). For each human antibody sequence of interest, we used the immunogenetics sequence analysis software Cloanalyst (https://www.bu.edu/computationalimmunology/research/software) to infer an unmutated common ancestor (UCA) sequence using only rhesus germline immunoglobulin gene segments. In this approach, Cloanalyst aligns the human antibody sequence to the closest rhesus V, D, and J gene segments using Cloanalyst**’**s rhesus Ig gene segment library which is based on a draft version of genome sequencing of the rhesus Ig loci (34). The resulting rhesus UCA is thus comprised of the rhesus V, D, and J gene segments with the highest identity to the human antibody sequence. Amino acids with high uncertainty in the inference of the rhesus UCA were changed to match the human amino acid at that position. The rhesus UCA amino acid sequence and the human amino acid sequence were then aligned, and CDR1, 2, and 3 of the rhesus UCA were replaced with the corresponding CDR1, 2, and 3 of the human amino acid sequence producing the rhesusized antibody sequence. The rhesusized v-regions for the heavy and light chain were attached to macaque constant regions (AF045537, AF050635, FJ795843).

### Antibody Expression and Purification

IgGs were prepared by co-transfection of heavy and light chain plasmids into HEK expi293F cells (Thermo Fisher Scientific) grown in expi293F expression medium in 8% CO_2_. Seven days post-transfection cells were pelleted by centrifugation and the medium filtered. IgGs were purified by passage of the medium over a HiTrap protein A column (GE Healthcare) equilibrated in PBS. IgGs were eluted with 0.1 M glycine pH 3.0 and the pH of the eluted protein immediately raised to neutral pH by addition of 1 M Tris-HCl pH 8.5. Fabs were generated by papain digest. IgGs were first incubated with immobilized papain (Thermo Fisher Scientific) at 37°C for 3-4 hours in 20 mM sodium phosphate pH 7.2 supplemented with 3.5 mg/ml cysteine. Immobilized papain agarose was removed by centrifugation and the supernatant filtered. Fabs were separated from undigested IgG and Fc by passage over a HiTrap protein A column. Fabs were then further purified by size exclusion chromatography over a Superdex 200 gel filtration column (GE Healthcare) equilibrated in 20 mM Tris-HCl pH 7.2 and 100 mM ammonium acetate.

### Preparation of Protein Complexes

Clade A/E 93TH057gp120 core_e_ with a His^375^ to Ser mutation was prepared by transfection of GnT1^-^ HEK 293F Freestyle cells (Thermo Fisher Scientific) with 0.5 mg of plasmid/liter of culture. Cells were grown in Freestyle 293 medium (Thermo Fisher Scientific) supplemented with 2.5% Ultra Low IgG Fetal Bovine Serum (FBS) (Gibco) in 8% CO_2_ for 7 days. Cells were pelleted, the medium filtered, and gp120 was purified from medium by passage over a 17b affinity column which consisted of the anti-gp120 antibody 17b covalently linked to protein A agarose. gp120 was eluted from the column with 0.1 M glycine pH 3.0 and the pH raised after elution by addition of 1 M Tris-HCl pH 8.5. The protein was concentrated and the buffer exchanged for 50 mM sodium acetate pH 6.0 and 300 mM sodium chloride. Glycans were then truncated by addition of Endo H_f_ (New England Biolabs) and incubation at 37°C overnight. Endo H_f_, endoglycosidase H linked to maltose binding protein, was removed by passage over an amylose column (New England Biolabs) equilibrated in 25 mM Tris-HCl pH 7.2 and 200 mM sodium chloride. The flow through fractions containing gp120 were further purified by size exclusion chromatography over a Superdex 200 gel filtration column (GE Healthcare) equilibrated in 20 mM Tris-HCl pH 7.2 and 100 mM ammonium acetate prior to use in complex formation with RhDH677.3 Fab. A V2 peptide corresponding to the clade A/E 92TH023 gp120 V2 loop sequence (Asp^167^-Lys-Lys-Gln-Lys-Val-His-Ala-Leu-Phe-Tyr-Lys-Leu-Asp-Ile-Val-Pro-Ile^184^) was synthesized by Genescript (www.genescript.com) without modifications for use in complex formation with RhDH827 Fab.

The C1C2 Cluster A region RhDH677.3 complex was made by mixing RhDH677.3 Fab with purified clade A/E 93TH057gp120 core_e_ and the CD4 mimetic M48U1 in a molar ration of 1.2:1.2:1 of Fab:M48U1:gp120. The complex was allowed to incubate on ice for 30 minutes before purification by size exclusion chromatography over a Superdex 200 gel filtration column equilibrated in 20 mM Tris-HCl pH 7.2 and 100 mM ammonium acetate. The V1V2 region RhDH827 complex was made by mixing RhDH827 Fab with V2 peptide resuspended in water in a molar ratio of 1.2:1 peptide:Fab. The complex was incubated on ice for 30 minutes and purified by size exclusion chromatography over a Superdex 200 column equilibrated in 20 mM Tris-HCl pH 7.2 and 100 mM ammonium acetate. In both cases elution fractions corresponding to the complex molecular weight were combined and concentrated to approximately 10 mg/ml for use in crystallization trials.

### Surface Plasmon Resonance Affinity Measurements of IgGs to Antigens

All surface plasma resonance (SPR) assays of IgGs to antigens were performed on a Biacore 3000 (GE Healthcare) with a running buffer of 10 mM HEPES pH 7.5 and 150 mM NaCl supplemented with 0.05% Tween 20 at 25° C. Kinetic measurements were done by immobilizing IgG on a protein A chip (Cytiva) and passage of serial dilutions of antigen in running buffer over the chip for 200 seconds. Complexes were then allowed to dissociate for 400 seconds by passage of running buffer at the same flow rate. IgG was removed from chip using regeneration buffer, 100 mM glycine pH 2.5, and fresh IgG reapplied between cycles. The antigen for C1C2 Cluster A region RM JR4, DH677.3, and RhDH677.3 was monomeric full length single chain (FLSC), a covalent dimer of gp120_BaL_ and CD4 that specifically exposes CD4i epitopes (35), in a concentration range of 6.25 to 200 nM and IgGs were immobilized on the protein A chip to a response unit (RU) of ~60-120. The antigen for gp41 region 7B2 and Rh7B2 was a synthetic gp41 peptide (residues 596-606) with C-terminal amidation (GenScript) in a concentration range of 6.4-25.6 μM and IgGs were immobilized to a RU of ~400-500. The antigen for V1V2 region DH827, RhDH827, and RM DH614.2 was a synthetic V2 peptide (gp120 residues 167-184) in a concentration range of 12.5-50 μM and IgGs were immobilized to a RU of ~600. Sensorgrams were corrected by subtraction of the corresponding blank channel and buffer background and normalized to a Rmax of 100 RU for curve fitting purposes. Kinetic constants were determined using a 1:1 Langmuir binding model with the BIAevaluation software (GE Healthcare). Goodness of fit of the curves were evaluated by the Chi^2^ of the fit with a value below 3 considered as being acceptable (based upon use of a Rmax = 100 RU).

### Crystallization, Data Collection and Structure Solution

Crystals were initially grown from commercial crystallization screens (Molecular Dimensions Proplex Eco and Rigaku precipitant synergy) and later optimized to produce crystals suitable for data collection by the hanging drop vapor diffusion method. C1C2 Cluster A region RhDH677.3 complex crystals were grown from 20% PEG 4000 and 0.1 M sodium citrate pH 4.5 and V1V2 region RhDH827 complex crystals were grown from 16.75% PEG 3350, 10.05% isopropanol, and 0.2 M ammonium citrate/citric acid pH 4.5. Crystals were briefly soaked in crystallization buffer supplemented with 20% MPD (2-Methyl-2,4-pentanediol) and flash frozen in liquid nitrogen prior to data collection.

Diffraction data for the RhDH677.3 complex were collected at the Stanford Synchrotron Radiation Light Source (SSRL) beamline 12-2 on a Dectris Pilatus 6M area detector and diffraction data for the RhDH827 complex were collected at the National Synchrotron Light Source II (NSLS-II) beamline 17-ID-2 on a Dectris Eiger 16M area detector. All data were processed and reduced with HKL2000 (36) or imosflm and scala from the CCP4 suite (37). Structures were solved by molecular replacement with PHASER from the CCP4 suite (37) based on the coordinates of the human DH677.3 complex for the RhDH677.3 complex (PDB ID 6MFP) and the macaque Fab structure of RM JR4 for the RhDH827 complex (PDB ID 4RFE). Refinement was carried out with Refmac (37) and/or Phenix (38) and model building was done with COOT (37). Data collection and refinement statistics are shown in **Table 1**.

**Table 1:** Data collection and refinement statistics.

	RhDH677.3 Fab-M48U1-gp120_93TH057_ core_e_	RhDH827 Fab-V2 peptide
**Data collection**		
Wavelength, Å	0.979	0.979
Space group	P2_1_	C2
Cell parameters		
a, b, c, Å	99.2, 82.7, 111.9	81.6, 71.9, 87.7
α, β, γ, °	90, 112.0, 90	90, 111.5, 90
Complexes/a.u.	2	1
Resolution, (Å)	50-2.9 (2.95-2.9)	50-2.0 (2.1-2.0)
# of reflections		
Total	77,728	126,613
Unique	31,091 (1,645)	31,034 (4,384)
R_merge_ ^a^, %	10.6 (62.7)	9.5 (47.4)
R_pim_ ^b^, %	7.8 (46.7)	5.3 (26.5)
*CC_1/2_* ^c^	0.98 (0.71)	0.99 (0.81)
Wilson B_factor_ (1/Å^2^)^d^	64	27.6
I/σ	9.6 (1.1)	5.3 (1.5)
Completeness, %	82.7 (86.0)	99.5 (98.2)
Redundancy	2.5 (2.5)	4.0 (4.0)
**Refinement Statistics**		
Resolution, Å	50.0 - 2.9	50.0 - 2.0
R^e^, %	25.5	18.1
R_free_ ^f^, %	29.6	20.9
# of atoms		
Protein	11,758	3,361
Water	15	273
Ligand/Ion	284	7
Overall B value (Å)^2^		
Protein	71	37
Water	42	43
Ligand/Ion	69	61
Root mean square deviation		
Bond lengths, Å	0.007	0.007
Bond angles, °	1.6	0.9
Ramachandran^g^		
favored, %	80.2	96.1
allowed, %	13.9	3.0
outliers, %	5.9	0.9
PDB ID	7N8Q	7N0X

Values in parentheses are for highest-resolution shell.

a R_merge_ = ∑|I - <I>|/∑I, where I is the observed intensity and <I> is the average intensity obtained from multiple observations of symmetry-related reflections after rejections.

b R_pim_ = as defined in (39).

c CC_1/2_ = as defined by Karplus and Diederichs (40).

d Wilson B_factor_ as calculated in (41).

e R = ∑||F_o_|- | F_c_||/∑|F_o_ |, where F_o_ and F_c_ are the observed and calculated structure factors, respectively.

f R_free_ = as defined by Brünger (42).

g Calculated with MolProbity.

### Structure Validation and Analysis

Ramachandran statistics were calculated with MolProbity and illustrations were prepared with Pymol Molecular graphics (http://pymol.org). The processed data and final model for the RhDH677.3 complex are deposited in the PDB with accession number 7N8Q and those for the RhDH827 complex are deposited with accession number 7N0X.

### Surface Plasmon Resonance Affinity Measurements of FcγR Binding

The affinity of IgG variants for RM and human Fcγ receptors (FcγRs) were measured as previously described (23). Briefly, the antibodies were immobilized on a medium density carboxymethyldextran sensor (Xantec Bioanalytics, CMD200M) using a Continuous Flow Microspotter (Carterra) and carbodiimide chemistry. The soluble analyte consisted of dilutions of receptor beginning at 20 μM and diluted by 1:3 over an 8-point series. Association and dissociation were measured for 5 minutes each on an imaging-based surface plasmon resonance (SPRi) instrument (IBIS Technologies, MX96). The results were analyzed in Scrubber 2 (BioLogic Software) using a first-order kinetic model to determine the equilibrium dissociation constant.

### HIV-1 IMC-Infection of CEM.NKR_CCR5_ Cells

The infection of cells was performed as previously reported (43). Briefly, R5 tropic HIV-1 IMC virus stocks (1086.C, CH505, SF162) were titrated to determine the input required for optimal viral gene expression within 72 h post-infection of CEM.NKR_CCR5_ cells as measured by intra-cellular p24 expression. Stocks were used to infect 2 × 10^6^ cells with each IMC by incubation with the appropriate dose for 30 min at 37°C and 5% CO_2_ in the presence of DEAE-Dextran (7.5 μg/mL). The cells were subsequently resuspended at 0.5 × 10^6^/mL and cultured for 2 days in complete medium containing 7.5 μg/mL DEAE-Dextran. On assay day, the infection was monitored by measuring the frequency of cells expressing intracellular p24. The assays performed using the IMC-infected target cells were considered reliable if the percentage of viable p24+ target cells on assay day was ≥20%. Assay data generated using infected cells was normalized to the % of target cells positive for intracellular p24.

### SHIV-Infection of A66 Cells

The infection of A66 cells [SupT1 cells (non-BC7 variant variant (44) that have been stably transfected to express both rhesus CD4 and rhesus CCR5 receptors after knockout of endogenous human CXCR4 and CD4 (45), provided by James Hoxie, University of Pennsylvania, Philadelphia, PA] was conducted as described previously (46). Briefly, R5 tropic SHIV virus stocks SF162.P3 (32) grown in human PBMCs, or CH505.375H (47) and 1157(QNE)Y173H (48) grown in Rhesus PBMCs were titrated to determine the input required for optimal viral gene expression within 72 h post-infection of A66 cells as measured by intracellular p27 expression. A66 cells (1 × 10^6^ cells per infection) were incubated for 4 hours at 37°C and 5% CO_2_ in the presence of DEAE-Dextran (10 μg/mL, Sigma Aldrich). The cells were subsequently resuspended at 0.33 × 10^6^/mL and cultured for 3 days in complete medium containing 10 μg/mL DEAE-Dextran. On assay day, infection was monitored by measuring the frequency of cells expressing intracellular p27. The assays performed using the SHIV-infected target cells were considered reliable if the percentage of viable p27+ target cells on assay day was ≥10%. Assay data generated using infected cells was normalized to the frequency of live target cells positive for intracellular p27.

### Infected Cell Antibody Binding Assay (ICABA)

ICABA was used to evaluate the ability of mAbs to bind Env on the surface of HIV- or SHIV-infected cells. HIV-infected CEM.NKR.CCR5 cells and SHIV-infected A66 cells were obtained as described above. Cells incubated in the absence of virus (mock infected) were used as a negative control. Infected and mock infected cells were washed in PBS, dispensed into 96-well V-bottom plates at 2 x 10^5^ cells/well and incubated with 1 μg/mL of indicated mAbs for 2 hours at 37 °C. After two washes with 250 μL/well WB, the cells were stained with vital dye (Live/Dead Fixable Aqua Dead Cell Stain, Invitrogen) to exclude nonviable cells from subsequent analysis. Cells were washed with wash buffer (5%FBS in PBS) and stained with anti-CD4-PerCP-Cy5.5 (clone OKT-4 for CEM.NKR.CCR5 cells or clone Leu-3 for A66 cells; BD Biosciences) to a final dilution of 1:20 in the dark for 20 min at room temperature (RT). Cells were then washed again, and permeabilized using Cytofix/Cytoperm (BD Biosciences). Anti-p24 antibody (clone KC57-RD1; Beckman Coulter, 1:100 dilution in 1x Cytoperm Solution, BD Biosciences) and a secondary FITC-conjugated antibody (goat anti-human IgG(H+L)-FITC, KPL, final dilution of 1:100 or Goat Anti Rh IgG(H+L)-FITC, Southern Biotech, final dilution of 1:200) for CEM.NKR.CCR5 cells; anti-p27 antibody (WNPRC Immunology Services, 1:500 dilution in 1x Cytoperm Solution, BD Biosciences) and a secondary PE-conjugated antibody (goat anti-human Ig Fc-PE, eBioscience, San Diego, CA., final dilution of 1:400 or Goat Anti-Rh IgG(H+L)-PE, Southern Biotech, final dilution of 1:200) were added to each well and incubated in the dark for 25 min at 4°C. Cells were washed three times with Cytoperm wash solution and resuspended in PBS-1% paraformaldehyde. The samples were acquired within 24 hours using a BD Fortessa cytometer. A minimum of 50,000 total events was acquired for each analysis. Gates were set to include singlet and live events. Data analysis was performed using FlowJo 9.6.6 software (BD Biosciences). Final data represents the frequency of infected cells (Ab+p24+ or Ab+p27+) and FITC MFI or PE MFI of binding of IgG mAbs to HIV Env, after normalization by subtraction of the frequency or MFI observed for cells stained with the secondary antibody alone.

### *Renilla* Luciferase-Based ADCC Assay

The LucR-based ADCC assay was conducted as described by Pollara et al. (49). The day prior to the ADCC assay, cryopreserved PBMCs to be used as effectors in the assay were thawed in R10 [RPMI medium supplemented with 10% Fetal Bovine Serum (FBS)], counted and assessed for viability and resuspended in R10 overnight. On the day of the assay, infected CEM.NKR_CCR5_ cells were counted, assessed for viability (viability was ≥ 80% to be used in the assay) and the concentration was adjusted to 2 x 10^5^ viable cells/mL (5 x 10^3^ cells/well). PBMCs were then counted, assessed for viability, pelleted and resuspended in the infected CEM.NKR_CCR5_ cells at a concentration of 6 x 10^6^ PBMCs/mL (1.5 x 10^5^ PBMCs/well) (effector: target cell ratio of 30:1). The mAbs starting at 50 µg/mL were serially diluted 1:5. The effector/target cell mix and antibody dilutions were plated in opaque 96-well half-area plates, centrifuged at 300 x g for 1 min after 30 min incubation at room temperature, and then incubated for 5.5 hrs at 37°C, 5.5% CO_2_ to allow ADCC-mediated cell lysis to proceed. After 5.5 hrs, ViviRen substrate (Promega) was diluted 1:500 in R10 and added 1:1 to the assay wells. The substrate generates luminescence only in live, infected cells; not in dead or lysed cells. The final readout was the luminescence intensity generated by the presence of residual intact target cells that have not been lysed by the effector population in the presence of ADCC-mediating antibodies. The percentage of specific killing was calculated using the formula


$$\%\text{ specific killing}=\frac{\text{RLU of target}+\text{effector well}-\text{RLU of test well }}{\text{RLU of target}+\text{effector well}}\times 100$$

In the analysis, the RLU of the target + effector wells represent lysis by effector cells in the absence of any source of antibody. Synagis, a human mAb specific for respiratory syncytial virus (50), and DSP_Rh, a macaque mAb specific for desipramine (DSPR1) from the nonhuman primate reagent resource (www.nhpreagents.org), were used as negative controls.

### ADCC-GranToxiLuc Assay

ADCC activity was detected according to the previously described ADCC-GranToxiLux (GTL) procedure using recombinant SHIV.1157QNE(Y173H) gp120 coated CEM. NKR_CCR5_ as target cells and cryopreserved PBMC from a HIV-seronegative donor as effector cells (51) with E:T ratio of 30:1 The mAbs starting at 50 µg/mL were serially diluted 1:5. The results of the GTL assay were considered positive if % Granzyme B activity after background subtraction was ≥8% for the infected target cells.

### ADCC AUC

The specific killing activities in the ADCC assay were summarized for each subject and antigen by computing the area under the dilution curve (AUC) which was calculated from dilution curves using non-linear trapezoidal rule. Non-specific killing activities mediated by negative control mAbs, Synagis or DSP_Rh, were subtracted from the corresponding human or rhesus mAbs prior to AUC calculation.

#### Antibody Dependent Cellular Phagocytosis (ADCP) and Antibody Dependent Neutrophil Phagocytosis (ADNP)

ADCP was performed as previously described (52, 53). Briefly, ADCP was quantified by covalently binding biotinylated SHIV 1157(QNE)Y173H gp120 Env glycoprotein (1µg gp120/µL of bead) to 9x10^5^ neutravidin fluorescent beads (ThermoFisher, F8776). Beads were then incubated with monoclonal antibodies for 2 hours to form immune complexes. THP-1 cells (ATCC, TIB-202) pre-treated with anti-CD4 (Biolegend, 344602) were then added to the immune complexes (25,000 cells/well) and spinoculated at 4°C. Following spinoculation, the cells were incubated at 37°C for 1 hour and fixed with 2% paraformaldehyde. The fluorescence of the cells was detected using flow cytometry (BD LSRFortessa). To calculate phagocytosis scores a cutoff was first assigned based on the 95th percentile of the no-antibody control (PBS only). The magnitude of the ADCP immune response (ADCP score) was calculated by multiplying the mean fluorescence intensity (MFI) and frequency of phagocytosis-positive cells and dividing by the MFI and frequency of the bead-positive cells in an antibody-negative (PBS) control well. Control antibodies were selected based positive binding to SHIV 1157(QNE) gp120 antigen. CH31_IgG3 mAb (Catalent), and CH235_12_IgA (54) (kindly provided by Huaxin Liao, Mattia Bonsignori, and Bart Haynes, Duke University) HIV specific, CD4bs broadly neutralizing antibodies (55), were included as positive controls and the non–HIV-specific CH65 IgG1 mAb (Catalent) (56) was included as a negative control. ADNP was performed as described for ADCP with the following modification: HL-60 cells (ATCC, CCL-240) pre-treated with DMSO for 5 days (57) were used as effector cells. The IgA antibody positive control was utilized to confirm that differentiation of the HL-60 cells to be neutrophil-like with expression of FcR alpha engagement. Positivity cutoff was 2.2 calculated from the mean + 3 standard deviations of the negative control.

#### Infectious Virion Capture Assay (IVCA) (Column)

Infectious virion capture assay (IVCA) was measured as previously described (53, 58–60). Briefly, the IVCA method utilizes a Protein G column based capture of Ig-virion immune complexes allowing for the analysis of infectious virions (TZM-bl infectivity assay). Briefly, antibodies were mixed with SHIV1157(QNE)Y173H (kindly provided by Abraham Pinter, Rutgers New Jersey Medical School) at final concentration of 25 μg/ml (200 μl volume) to form Ab-virion immune complexes (IC), which were passed through a protein G column. The infectivity of the flow-through was measured by a TZM-bl infection assay. The percentage of captured infectious virions (iVirion) were calculated as follows: iVirion = [(100- flow-through infectivity)/(virus no-Ab infectivity)] x 100. HIVIG polyclonal human sera (NIH HIV Reagent Program) was included as a positive controls and the non–HIV-specific CH65 IgG1 mAb (Catalent) (61) was included as a negative control. Positivity cutoff was 15.6% calculated from the mean + 3 standard deviations of the negative control.

## Results

### Engineering Rhesusized Variants of Human mAbs

**Figure 1:**
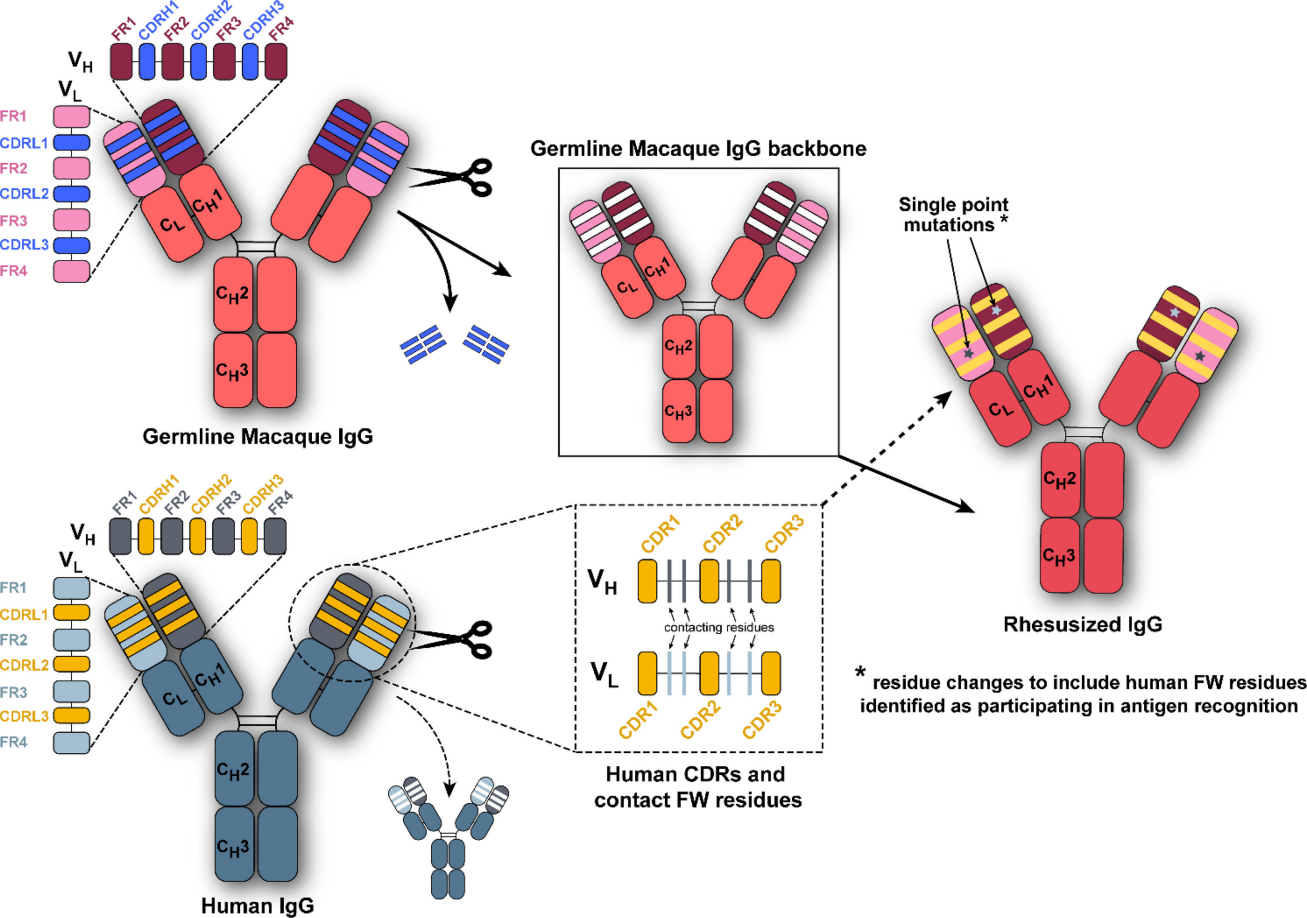
Composition scheme for rhesusized antibody engineering. We grafted the human complementary-determining regions (CDRs) and any framework (FW) residues contributing to antigen binding when structural data was available of the parental human monoclonal antibody (bottom left panel) onto the macaque germline framework and antibody backbone (top left panel) to generate the rhesusized antibody (right panel).

Three HIV-1 specific human antibodies, DH677.3, DH827, and 7B2, were converted to macaque versions, i.e. rhesusized, with the aid of both the macaque genome and structural information about each antibody’s epitope (30–33). For clarity rhesusized versions of antibodies will begin with an Rh in the name and native macaque antibodies will have an RM designation; human antibodies will have no designation to be consistent with the names assigned to them by the literature. Sequence changes were kept to a minimum to maintain specificity and reduce immunogenicity. The schematic of how theses rhesusized variants were made is shown in **Figure 1**. First, the inferred macaque germline gene closest in sequence to the human antibody was identified. At positions where the equivalent position in the macaque germline sequence could not be determined the corresponding amino acid from the human sequence was used. Complementary determining regions (CDRs) were then changed to that of the human antibody. When structural information about the epitope in human showed that residues outside of the CDRs were involved in binding antigen, those human contact residues were introduced into the macaque germline sequence. The rhesusized antibody therefore consists of macaque framework sequences modified to be human only at positions that are part of the paratope and human CDR sequences with fully macaque constant regions (the resulting rhesusized sequences are shown in **Figure S1**). Altogether, generation of Rh7B2 V_H_ and V_L_ required 18 changes in sequence for heavy chain framework residues and 17 changes in framework residues for the light chain relative to the human 7B2; one additional heavy chain change relative to the macaque sequence was required due to a framework epitope contact and one light chain change due to a missing macaque residue at position 65 in the germline sequence. Generation of RhDH677.3 V_H_ and V_L_ required 9 heavy chain and 7 light chain changes in sequence relative to the human DH677.3 as well as one heavy chain change at position 6 due to a missing residue in the macaque germline sequence. Generation of RhDH827 V_H_ and V_L_ required 11 heavy and 7 light chain framework changes in sequence. The heavy chain C_H_ (C_H_1) and Fc (C_H_2 and C_H_3) of all the antibodies were fully macaque with 27 changes and 3 inserted glycine residues relative to the human IgG1 (91% overall identity). C_L_ differences varied for each antibody depending on whether the light chain was kappa, RhDH677.3 and Rh7B2, or lambda, RhDH827. RhDH677.3 and Rh7B2 had 17 changes in their C_L_ kappa light chains relative to their human counterparts (84.1% identity) and RhDH827 had 12 changes in its C_L_ relative to its lambda equivalent (88.6% identity). Therefore, excluding CDRs, the Rh7B2 Fab was 78.4% identical in sequence to the 7B2 Fab and 98.8% identical in sequence to the inferred macaque germline gene, the RhDH677.3 Fab was 90.4% identical in sequence to the DH677.3 Fab and 99.4% identical to its macaque germline sequence, and the RhDH827 Fab was 88% identical to the DH827 Fab and 100% identical to its macaque germline sequence.

### Rhesusized mAb Variants Bind to RM and Human Fcγ Receptors With Similar Binding Affinities to Those of RM Origin

**Figure 2:**
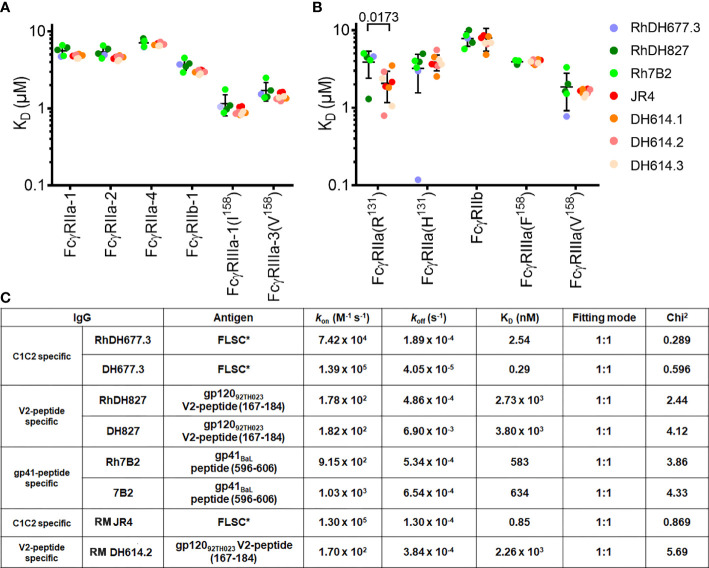
Affinity of rhesusized variants for low affinity receptors. Binding affinities of **(A)** rhesus and **(B)** human FcγRs. Equilibrium binding constants (K_D_) measured by SPR for common rhesus and human FcγR allotypes. Data show individual replicates, and error bars denote the standard deviation. Data are representative of 2 independent experiments. RhDH677.3, RhDH827, and Rh7B2 are rhesusized mAbs. RM JR4 (62), RM DH614.1, RM DH614.2, and RM DH614.3 (63) are mAbs originally isolated from a RM vaccinated with the vaccine regime used in RV144. The P-value for an unpaired T-test for rhesusized and RM mAbs is shown above the compared pair. **(C)** SPR binding kinetics for rhesusized (RhDH677.3, RhDH827, and Rh7B2) and human [DH677.3 (30), DH827 (31) and 7B2 (32)] mAb pairs. RM JR4 (62) and RM DH614.2 (63), mAbs originally isolated from RM, were used as controls. FLSC, a covalent dimer of gp120_BaL_ and domain 1 and 2 of CD4 (35) was used as antigen for C1C2 Cluster A specific mAbs RhDH677.3, DH677.3 and control JR4, gp120_92TH023_ V2 peptide (residues 167-184) was used as antigen for V1V2 region mAbs RhDH827, DH827 and control RM DH614.2, and gp41_BaL_ peptide (residues 596-606) was used as antigen for gp41 region specific mAbs Rh7B2 and 7B2.

Proper rhesusization should result in an antibody variant that preserves its structural integrity and both the binding properties mediated by the Fc of the equivalent RM IgG subclass and those mediated by the Fab (i.e. epitope specificity and binding interface) of the parent human mAb. Accordingly, rhesusized mAb variants were generated in a RM IgG1 backbone to match the original parent human IgG1 backbone (**Figures 1** and **S1**). To validate this design we tested the Fc binding properties of our rhesusized variants to receptors expressed on effector cells involved in Fc-effector functions to see if they were comparable to mAb IgG1s of RM origin. We show in **Figure 2**, **Table S1** and **Figure S2** the SPR binding kinetics of RhDH677.3, Rh7B2 and RhDH827 to both rhesus and human low affinity FcγRs measured with IgG immobilized on a sensor chip and FcγR in solution. Two antibody specificities were used as controls of RM origin. The antibody used as a control for an epitope from the C1C2 Cluster A region was RM JR4, a mAb derived from the peripheral blood B cells of a rhesus macaque infected with the simian-human immunodeficiency virus (SHIV) KB9 mutant that contains gp41 glycosylation site deletions (62). Antibodies used as a control for an epitope in the V1V2 region were clonal variants of RM DH614 (RM DH614.1, RM DH614.2 and RM DH614.3), mAbs isolated from a RM vaccinated with the vaccine regimen used in RV144 (63). Among the receptors tested were polymorphic variants 1, 2 and 4 of RM FcγRIIa (e.g. RM FcγRIIa-1,-2 and -4), RM FcγRIIb-1 and RM FcγRIIIa-1 and 3 as described in (23). Human receptors tested included high and low affinity variants of FcγRIIa and FcγRIIIa and a low affinity inhibitory receptor FcγRIIb. Interestingly, binding affinities of the rhesusized IgG variants to all low affinity RM receptors tested were in the range of binding affinities detected for IgG variants that were cloned directly from an RM host with no statistical difference between the groups. The only statistical difference between the rhesusized and RM antibody groups was seen in an unpaired T-test for the low affinity allelic variant of human FcγRIIa(R131) with P value = 0.0173. No significant differences in affinities to the other human Fcγ receptors were detected This indicates that the engineered rhesus Fc backbone is properly folded and the variants fully preserve the Fc-binding properties of an IgG1 of RM origin to all RM Fcγ receptors involved in Fc-effector mechanisms and all but one human low affinity Fcγ receptors. The rhesusized mAb variants therefore preserve the RM IgG1 structure with variable regions (V) and antigen-binding characteristics of the parent human mAb. Rhesusized mAb variants bind to RM and human Fcγ receptors with similar binding affinities to those of RM origin.

### Rhesusized mAb Variants Bind to Antigen With Similar Binding Affinities to Those of Human Origin

We also examined the integrity and binding properties of the Fab to see if the recognition of HIV-1 Env was preserved in the variants as compared to their human counterparts. First, we used surface plasmon resonance analyses (SPR) to test the biding properties of human and rhesusized antibody pairs to appropriate antigens. For CD4 inducible (CD4i) antibodies (e.g. RM JR4 and DH677.3) we used full length single chain (FLSC), a covalent dimer of gp120_BaL_ and CD4 that specifically exposes CD4i epitopes (35). For antibodies specific for linear epitopes within the V2 loop (e.g. DH827 and D614 lineage) or gp41 (e.g. 7B2) we used the peptides that were used in co-crystallization studies (30, 32). As shown in **Figure 2B** and **Figure S3** all pairs tested with the exception of DH677.3 had identical or highly similar binding kinetics; RhDH677.3 had an approximately 8-fold lower K_D_ than human DH677.3 mainly due to an increase in the off-rate. This difference in affinity could be result of slight differences in the paratope, discussed in more detail below, or the result of removal of a glycan attached to Asn^72^ of the heavy chain sequence (Asp^72^ in RhDH677.3); this glycan sits outside of the paratope but could affect the affinity by interacting with the gp120 N-terminus.

**Figure 3:**
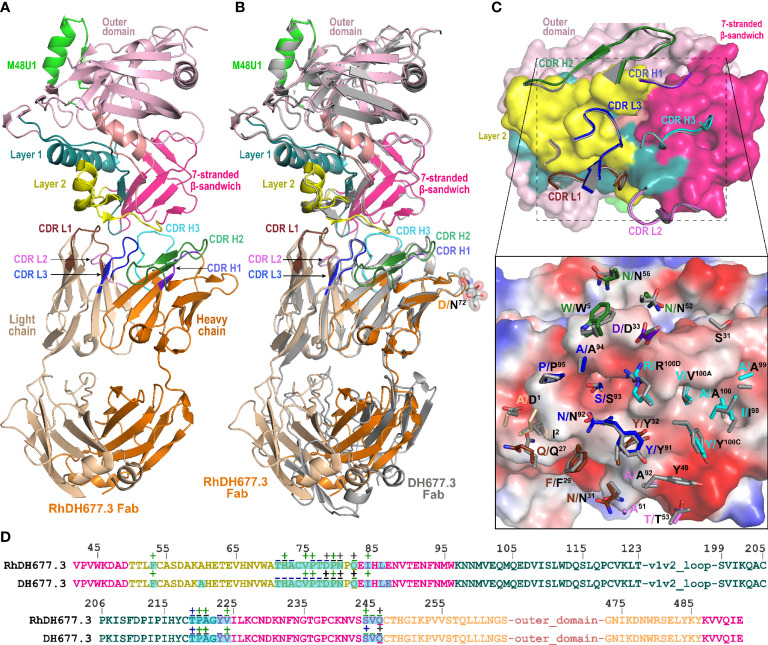
Crystal structure of RhDH677.3 Fab-gp120_93TH057_ core_e_-M48U1 complex. **(A)** The overall structure of the complex is shown as a ribbon diagram. The light chain (LC) and heavy chain (HC) of the RhDH677.3 Fab are colored in wheat and orange, respectively. The complementary-determining regions (CDRs) of RhDH677.3 Fab are colored as following: CDR H1 is purple blue, CDR H2 is dark green, CDR H3 is cyan, CDR L1 is dark brown, CDR L2 is purple, and CDR L3 is blue. Outer domain of gp120 is light pink. Layers 1, 2 and the 7-stranded -sandwich of the inner domain are colored as green, yellow and magenta, respectively. **(B)** Structural comparison of RhDH677.3 Fab-gp120_93TH057_ core_e_ and DH677.3 Fab-gp120_93TH057_ core_e_ complexes. The RhDH677.3 Fab-gp120_93TH057_ core_e_ complex is colored as indicated in panel A and the DH677.3 Fab-gp120_93TH057_ core_e_ complex is colored in gray. The complexes are superimposed based upon gp120 and the heavy and light chain variable (V_H_ and V_L_) domains. The carbohydrate at position Asn^72^ of the DH677.3 Fab is shown as sticks. **(C)** The RhDH677.3 Fab-gp120_93TH057_ core_e_ interface. gp120 is shown as a molecular surface and colored as described in A (top panel) and by electrostatic potential (bottom panel) with red, blue and white representing negative, positive and neutral electrostatic potential, respectively. CDRs of RhDH677.3 Fab are shown as ribbons (top panel) and side chains of binding residues are shown as sticks (bottom panel). Binding residues of RhDH677.3 Fab are colored as in panel A and binding residues of DH677.3 Fab are colored in dark gray. **(D)** Contact residues of gp120 are mapped onto the gp120_93TH057_core sequence. Contact residues are defined by a 5 Å cutoff and marked above the sequence with (+) for side chain and (-) for main chain to indicate the type of contact. Contact types are colored as following: hydrophilic (green), hydrophobic (blue) and both (black). Buried surface residues are as determined by PISA and are shaded green.

Next, to more carefully examine the molecular details of the antibody-antigen interface and to assess if mAb epitopes were fully preserved in the rhesusized variants we solved crystal structures of both the RhDH677.3 and RhDH827 Fabs in complex with their respective cognate Env antigens (**Table 1**). Shown in **Figure 3** is the structure of RhDH677.3 Fab in complex with gp120_93TH057_core_e_ and the CD4 peptide mimetic M48U1. The parent of RhDH677.3, DH677.3, is a Cluster A specific antibody isolated from a RV305 vaccinee, a follow up study of a subset of RV144 vaccinees with delayed boosting using RV144 immunogens (31, 64) with a similar epitope footprint to RV144 mAbs (65). The RhDH677.3 Fab-gp120_93TH057_ core_e_-M48U1 complex (**Figure 3A** **)** crystallized in space group P2_1_ with two complexes in the asymmetric unit and diffracted to 2.9 Å resolution (**Table 1**). The CD4 mimetic M48U1 was only found bound to one of the two complexes in the asymmetric unit due to clashes with neighboring complexes in the crystal. The gp120 in the second complex was therefore more disordered and less like the typical CD4-bound gp120 conformation present in the first complex of the asymmetric unit. However, within the region corresponding to the antibody-gp120 interface both copies of the complex were still highly similar with nearly identical conformations (**Figure S4**). Since the RV305 mAb DH677.3 also crystallized with two complexes in the asymmetric unit but with M48U1 bound for both copies (31), we only used the first complex (with M48U1 bound) from the RhDH677.3 Fab-gp120_93TH057_ core_e_-M48U1 complex crystal structure for comparison (**Table S2**). Values from the DH677.3 structure on the other hand, represent an average of those from both copies.

**Figure 4:**
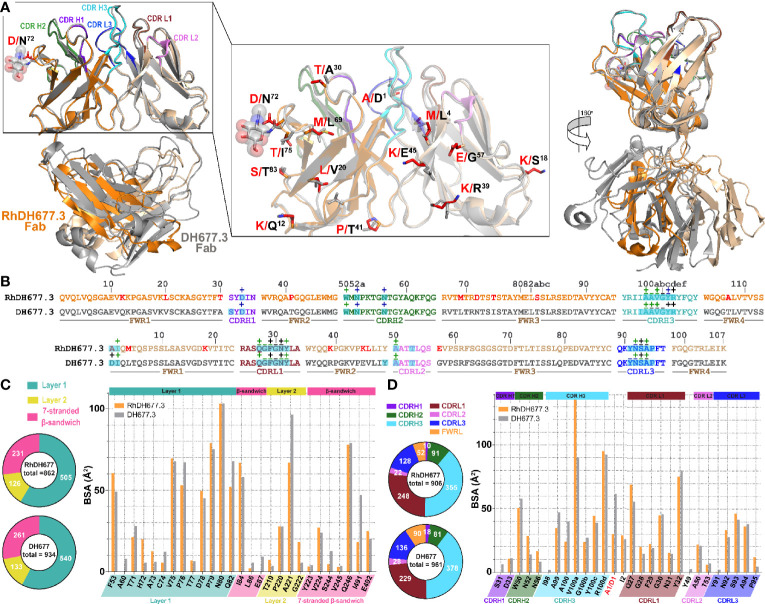
Structural comparison of RhDH677.3 and DH677.3 Fab antigen complexes. **(A)** The RhDH677.3 Fab and its CDRs are colored as indicated in **Figure 3** and the DH677.3 Fab is colored in gray. The complexes are superimposed based on the variable domains of light and heavy chains. Residues that differ between the two Fab are shown in stick and highlighted in red for RhDH677.3 Fab and in black for DH677.3 Fab (middle panel). A 180˚ view reveals the structural difference of the constant domains in RhDh677.3 and DH677.3 Fabs (right panel) **(B)** Contact residues of RhDH677.3 and DH677.3 Fabs with gp120 are mapped onto the Fab sequences and colored as described for panel **(A)** Contact residues are defined by a 5 Å cutoff and marked above the sequence with (+) for side chain and (-) for main chain to indicate the type of contact. Contact types are colored as following: hydrophilic (green), hydrophobic (blue) and both (black). Residues that differ between RhDH677.3 and DH677.3 are highlighted in red. Buried surface residues are determined by PISA and are shaded green. **(C)** Pie charts showing the buried surface binding (BSA) contributions of gp120 to binding and are colored as in **Figure 3** . The BSA contributions to binding of gp120 residues are shown as calculated by PISA. BSA contributions for gp120 contact residues to RhDH677.3/DH677.3 binding are shown in orange and gray, respectively. **(D)** Pie charts showing the buried surface binding (BSA) contributions to gp120 binding for RhDH677.3/DH677.3 Fabs and are colored as in panel **(A)** The BSA contributions to binding for RhDH677.3 CDR residues are colored in orange and BSA contributions to binding for DH677.3 CDR residues are shown in gray.

**Figure 5:**
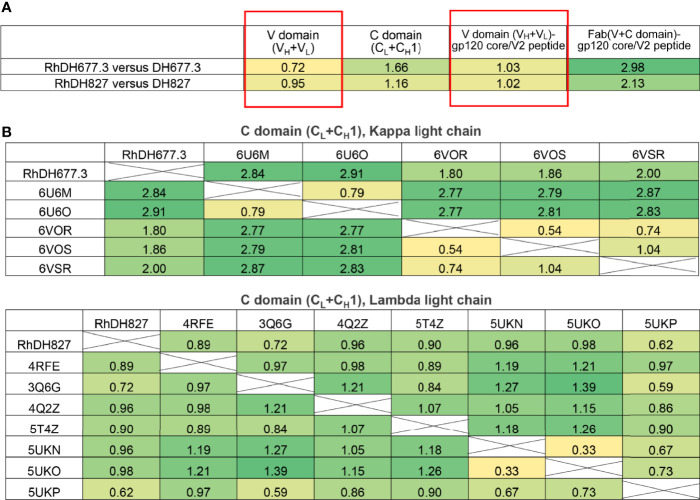
Comparison of overall structures of Rhesusized mAb variants to human and RM counterparts. **(A)** RMSD values for main chain atoms for pairwise comparisons of Fab V or C domains and Fab V or Fab-gp120 core complex between rhesusized and human variants. **(B)** RMSD values for main chain atoms for pairwise comparisons of Fab C domains of RhDH677.3 and RhDH827 to RM mAb Fabs available in the PDB with kappa and lambda light chains, respectively.

RhDH677.3, similar to its progenitor DH677.3, binds at the base of the 7-stranded β-sandwich of the gp120 inner domain with contributions from the inner domain mobile layers 1 and 2 **(** **Figure 3** **).** The total buried surface area (BSA) for the RhDH677.3-gp120 interface in the complex is 1800 Å^2^ which is very similar to the BSA for the interface contributed by its human counterpart, DH677.3, 1894 Å^2^ (**Table S2**). RhDH677.3 antibody-gp120 contacts involve residues in layer 1 (residues 53, 71-80 and 82), layer 2 (residues 219-222), and the 7-stranded β-sandwich (residues 84, 223-224, 244-246, and 491-492) (**Figure 3C**) almost entirely mimicking the antigen footprint of DH677.3. The only noticeable difference with its human counterpart is that the RhDH677.3 Fab-gp120 interface lacks two additional gp120 residues buried in the DH677.3 Fab-gp120 interface, i.e. Ala^60^ and Glu^87^. Overall, the lower total BSA for the macaque RhDH677.3 complex is mostly a result of slightly lower BSA values in most gp120 regions (**Figures 3B–D**, **Table S2**). The macaque complex buries 530 Å^2^ of the gp120 inner domain layer 1, 120 Å^2^ of layer 2, and 226 Å^2^ of the 7-stranded β-sandwich, while the human structure buries an average of 540 Å^2^ of layer 1, 133 Å^2^ of layer 2, and 261 Å^2^ of the 7-stranded β-sandwich. The added surface area for the human complex comes in part from additional contributions from light chain framework contacts near the N-terminus, slightly higher BSA values for CDRs H3 and L3, and framework residue differences between the human and macaque antibodies (**Figure 4** and **Figure S2**). The framework residue changes include light chain framework region 1 which contains an alanine, Ala^1^, in the macaque germline sequence and an aspartic acid, Asp^1^, in the human sequence. Ala^1^ in the macaque sequence does not contact gp120 at all, while Asp^1^ *via* its side chain makes main chain contacts to gp120 residues Thr^71^, Cys^74^, Val^75^, and Pro^76^ and side chain contacts with Pro^76^ (**Figure 3C**),. This one residue difference largely accounts for the 31 Å^2^ increase in BSA for this region (**Figure 4C, D**). The second is in the heavy chain framework region 3 which contains an aspartic acid, Asp^72^, in the macaque germline sequence that is not glycosylated but an asparagine, Asn^72^, in the human structure that is. This glycan sits outside of the antibody paratope on the edge of the β-sandwich and is not directly involved in binding in the 7-stranded β-sandwich gp120 complex but could potentially make contact to the eighth strand in an 8-stranded β-sandwich complex. One additional framework residue difference seems to have an indirect effect on the interface. In the macaque structure Ser^31^ in CDR H1 does not contribute to the interface, but in human structure Ala^30^ instead of Thr^30^ in framework region 1 allows Ser^31^ to contribute to the interface and the total BSA. In total these sequence differences help explain the 58 Å^2^ greater gp120 BSA and the 36 Å^2^ greater Fab BSA for the human complex as compared to the macaque complex. Despite the differences in the BSA values, the gp120 antigen complexes formed by both antibodies are highly similar with root mean square deviations (RMSD) between main chain atoms of the variable domains of the Fab and the variable domains of the Fab-gp120 core interface of 0.72 and 1.03 Å^2^, respectively (**Figure 5** **).** Altogether the structural analysis confirms the close similarity between RhDH677.3 and DH677.3 with regard to their recognition of gp120, although sequence differences within framework regions do modulate the binding interface even when they only make minor contributions, either directly like Ala^1^ in the macaque light chain or indirectly like Thr^30^ in the macaque heavy chain.

The second rhesusized mAb included for structural characterization was DH827, an antibody from the RV144 vaccine trial (30). In contrast to DH677.3 which is specific for a discontinuous, conformational epitope, DH827 recognizes a linear epitope within the V2 loop region. Interestingly, V2 loop specific antibodies fall into four main categories depending upon the V2 loop conformation and epitope region (66). V2qt and V2q antibodies bind the V2 loop in the quaternary trimeric form of the envelope structure (V2qt) such as PGT145 or (V2q) such as PG9 and PG16. V2i antibodies, e.g. 830A, directly overlap the α_4_β_7_ integrin binding site on the V2 loop, and V2p antibodies bind the V2 loop in peptide-like conformation that only partially overlaps the integrin binding site, e.g. RV144 mAbs CH58, CH59, and DH827 (67–69). A sieve analysis of breakthrough HIV-1 infections in RV144 identified Lys^169^ as a site of antibody induced pressure on the virus (7). V2p antibodies from RV144 vaccinees (including DH827) often contain a Glu-Asp motif in their CDR L2 and depend on Lys^169^ in binding the V2 loop (70). DH827 differs from many other V2p Abs in that it is less dependent upon Lys^169^.

**Figure 6:**
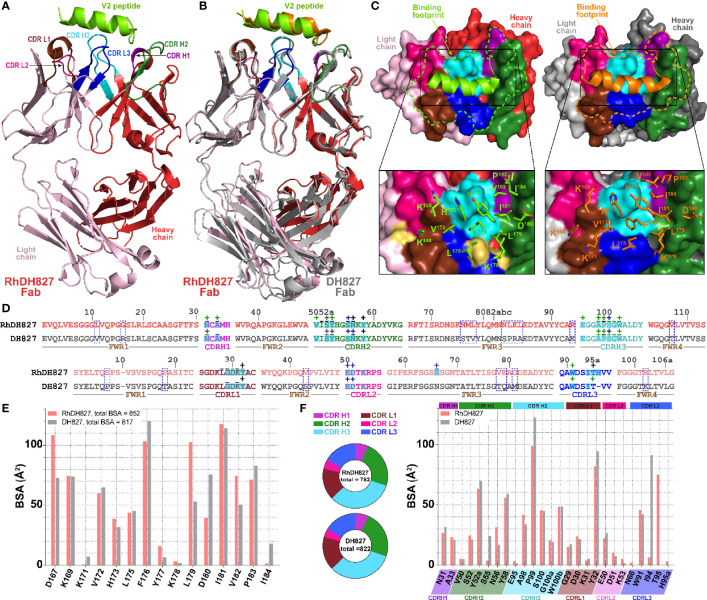
Crystal structure ofRhDH827 Fab-V2 peptide complex. **(A)** The overall structure of the complex is shown as a ribbon diagram. The V2 peptide is green and the light chain (LC) and heavy chain (HC) of RhDH827 Fab are colored in light pink and red, respectively. The CDRs of RhDH827 Fab are colored as following colors: CDR L1 (brown), CDR L2 (pink), CDR L3 (blue), CDR H1 (purple), CDR H2 (dark green), and CDR H3 (cyan). **(B)** Structural comparison of RhDH827 Fab-V2 peptide and DH827 Fab-V2 peptide complexes. The RhDH827 Fab-V2 peptide complex is colored as indicated in panel A and the DH677.3 Fab-V2 peptide complex is colored in gray with V2 peptide colored in orange. The complexes are superimposed based on the variable heavy (V_H_) domain. **(C)** RhDH827 andDH827 Fabs are shown as a molecular surface and the CDRs of both Fabs surface are colored as in panel **(A)** The light chain (LC) and heavy chain (HC) of DH827 Fab are colored in light and dark gray, respectively. Binding footprints for V2 peptide on RhDH827 and DH827 Fabs are outlined in green and orange, respectively (top panel). Residues contributing to the binding are shown as sticks (bottom panel). Extra binding residues on RhDH827 are colored in yellow. **(D)** Contact residues of RhDH827 and DH827 Fabs with V2 peptide are mapped onto the Fab sequences and colored as described for panel **(A)** Contact residues are defined by a 5 Å cutoff and marked above the sequence with (+) for side chain and **(-)** for main chain to indicate the type of contact. Contact types are colored as following: hydrophilic (green), hydrophobic (blue) and both (black). Residues that differ between RhDH827 and DH827 are highlighted in blue dashed-line box. Buried surface residues are determined by PISA and are shaded green. **(E)** The buried surface area (BSA) contributions of V2 peptide to binding of RhDH827/DH827 Fabs are shown in red and gray, respectively. The BSA contributions to binding of V2 residues as calculated by PISA. **(F)** Pie charts showing the BSA of RhDH827/DH827 Fabs buried at the complex interface and are colored as in panel **(A)** The BSA contributions to binding for RhDH827 CDR residues are colored in red and BSA contributions to binding for DH677.3 CDR residues are shown in gray.

In order to confirm that the rhesusized RhDH827 mAb maintains the V2p specificity of its parent mAb, we crystallized RhDH827 in complex with a clade A/E 93TH057gp120 V2 sequence peptide (**Table 1**, **Figure 6**). The crystals belonged to space group C2, diffracted to 2.0 Å resolution, and contained one RhDH827 Fab heavy and light chain bound to one V2 peptide in the asymmetric unit. RhDH827 binds the V2 peptide in a more helical conformation than many of the other RV144 Abs, e.g. CH58 and CH59, which allows it to make hydrophobic contacts with Val^172^, Leu^175^, Phe^176^, and Leu^179^. A discontinuation of the helix at the C-terminus allows it to extend its contacts to Ile^181^, Val^182^, and Pro^183^ (**Figures 6B, C**). Sixty-seven (67)% of the total 852 Å^2^ peptide BSA comes from these hydrophobic residues as opposed to 21.4% from Lys^168^ and Lys^169^ (**Figures 6D, E**). Outward facing residues of the helix contribute little if anything to the peptide BSA, i.e. Gln^170^, Lys^171^, Ala^174^, Tyr^177^, and Lys^179^. Likewise, RhDH827 is also less dependent upon Lys^169^. It focuses instead upon Lys^168^ and downstream hydrophobic residues in the V2 sequence; Lys^169^ accounts for 8.7% of the total peptide BSA while Lys^168^ accounts for 12.7% (**Figures 6C–E**). RhDH827 binds the V2 peptide mainly with CDRs H2, H3, L1 and L3. Glu^50^ and Asp^51^ of the Glu-Asp motif in CDR L2 make salt bridges with Lys^169^ and Lys^168^ of the peptide respectively, but account for almost all of CDR L2’s contribution to binding (**Figure 6F**). CDRs L1 and L3 provide much of the remaining contact surface for the N-terminus of the peptide, while CDRs H2 and H3 primarily interact with the C-terminus. The total light chain BSA contribution to the interface is 293 Å^2^, 124 Å^2^ from CDR L1, 37 Å^2^ from CDR L2, and 130 Å^2^ from CDR L3, and the total heavy chain BSA contribution 492 Å^2^, 51 Å^2^ from CDR H1, 184 Å^2^ from CDR H2, and 257 Å^2^ from CDR H3 (**Table S2**). Aside from the two salt bridges from CDR L2 (Glu^50^ and Asp^51^ to Lys^169^ and Lys^168^ respectively) and three hydrogen bonds from the heavy chain (one from CDR H3 Ser^100^ to His^173^ and two from CDR H2 tyrosine residues to the main chain carbonyls of Leu^179^ and Ile^181^) most of the interface is hydrophobic in nature which may help it tolerate mutations that disrupt the salt bridges to Lys^168^ or Lys^169^ and changes to Ile^181^, the hydrophobic residue with the single largest contribution to the BSA; changes in Lys^169^ and Ile^181^ were identified in RV144 breakthrough viruses implicating this region in the protective effect of the vaccine (7).

RhDH827 was crystalized with the same V2 peptide as used previously for its human counterpart DH827 (30) however, the complex structure of RhDH827 was solved at higher resolution (2.0 versus 2.9 Å for DH827). In addition, there is a one amino acid insertion in the light chain CDR L3 at position 95A of RhDH827 that is absent in DH827 due to a difference in sequence between the clone of DH827 used to generate RhDH827 (30). These differences slightly complicate the direct comparison of the two structures. Although the RMSD value for the comparison of the main chain atoms of the antibody variable domains and the bound peptide is very low, 1 Å (**Figure 5A**), there are some differences in specific contacts at the antibody-peptide interface. For example, in the RhDH827-V2 complex, Lys^168^ and Lys^169^ of peptide account for approximately 18% of the total peptide BSA with 8.9 Å^2^ and 9.1 Å^2^ contributed for Lys^168^ and Lys^169^, respectively. Furthermore, in the RhDH827 complex, the N-terminus of the peptide fold into an amphipathic helical conformation with hydrophobic residues from that region Val^172^, Leu^175^, Phe^176^, and Leu^179^ together with those from the C-terminus after a break in the helix e.g. Ile^181^, Val^182^, and Pro^183^, making up approximately 65% of the peptide BSA. This is comparable to the 67% seen for the corresponding residues in the DH827 complex, but the total peptide BSA is significantly lower for the human antibody, 817 Å^2^ versus 852 Å^2^. The lower resolution of the human complex could potentially explain this difference; density for the peptide is weaker at both the N and C termini in the human structure which makes the side chain positions for these residues less certain.

Interestingly, contributions from the heavy and light chain CDRs are roughly comparable between the two structures with the exception of one major difference in CDR L3. RhDH827 has an extra histidine, His^95A^, relative to the human DH827, due to a difference in the DH827 clone used to make RhDH827 (**Figure 6C** and **Figure S2**). This changes the conformation of the CDR L3 loop. As a consequence, CDR L3 Ile^94^ makes the same contacts to the peptide in the human complex that CDR L3 Thr^94^ makes to the peptide in the rhesusized complex. His^95A^ itself adds little to the interface. The slightly shorter CDR L3 in the human structure also changes the conformation of the C-t2erminus of the peptide, mainly in the area near the end of the peptide helix. This conformational difference may explain the slightly higher BSAs for CDR H3 Pro^99^ and CDR L1 Tyr^32^ in the human structure. Outside of these regions the structures are largely identical with similar BSA values for both the heavy and light chain residues as can be seen in the bar chart of BSA by residue (**Figure 6F**).

### Constant (C) Regions of Rhesusized mAb Variants Show Close Structural Similarity to C Domains of RM IgG1

Analysis of the structures of Fab-antigen complexes of rhesusized and human IgG pairs confirm close similarity of their V domain structures and good preservation of the contacts at the antigen-Fab interface. While in the rhesusized IgG1 variants the V domains are of mixed sequence, assembled from the closest RM germline framework mAb sequence and CDRs engrafted from human counterpart, the C domains (C_L_+C_H_1) are unchanged and formed from fully RM IgG1 sequences (**Figure 1**). Of note RhDH677.3 and RhDH827 represent kappa and lambda light chains, respectively. In order to check if sequence changes in the V domains contribute to changes in overall architecture of the rhesusized variant C domains we compared the structures of the C domains of RhDH677.3 and RhDH827 to C domain structures from RM IgG1 Fabs available in Protein Data Bank (**Figure 5B**). We used entries from PDB of antibodies of different specificities (including antibodies unrelated to HIV-1) but matched RhDH677.3 with antibodies with kappa light chains and RhDH827 with antibodies with lambda light chains. Overall the RMSD values for the C domains of RhDH677.3 as compared to the other kappa light chain containing antibodies fell within a range of 1.8-2.91 Å which is slightly higher than the highest RMSD value for kappa light chain antibody comparisons in the absence of RhDH677.3 (RMSD range of 0.54-2.87 Å). In contrast, the RMSDs for the C domains of RhDH827 as compared to the other lambda light chain containing antibodies fell within a range of 0.62-0.98 Å which is lower than the RMSD range for all other lambda antibodies in the absence of RhDH827 (0.33-1.39 Å). This indicates that there is a poorer agreement among the kappa light chain C domain structures in general and with RhDH677.3 in particular although this could be in part due to the lower resolution from the RhDH677.3 complex structure. The better agreement among the C domain structures with lambda light chains may be a reflection of the higher resolution of the RhDH827 structure but it could also be in part due to the fewer number of changes relative to the human sequence for the lambda light chain, 12, versus the kappa light chain, 17, since human lambda and kappa C domains were initially used as models for the generation of both structures.

### Rhesusized mAb Variants Mediate Antibody Fc Effector Functions (i.e. Recognition of SHIV-1 Infected Cells, ADCC, ADCP, ADNP, and Virion Capture) Comparable to Their Human Counterparts

**Figure 7:**
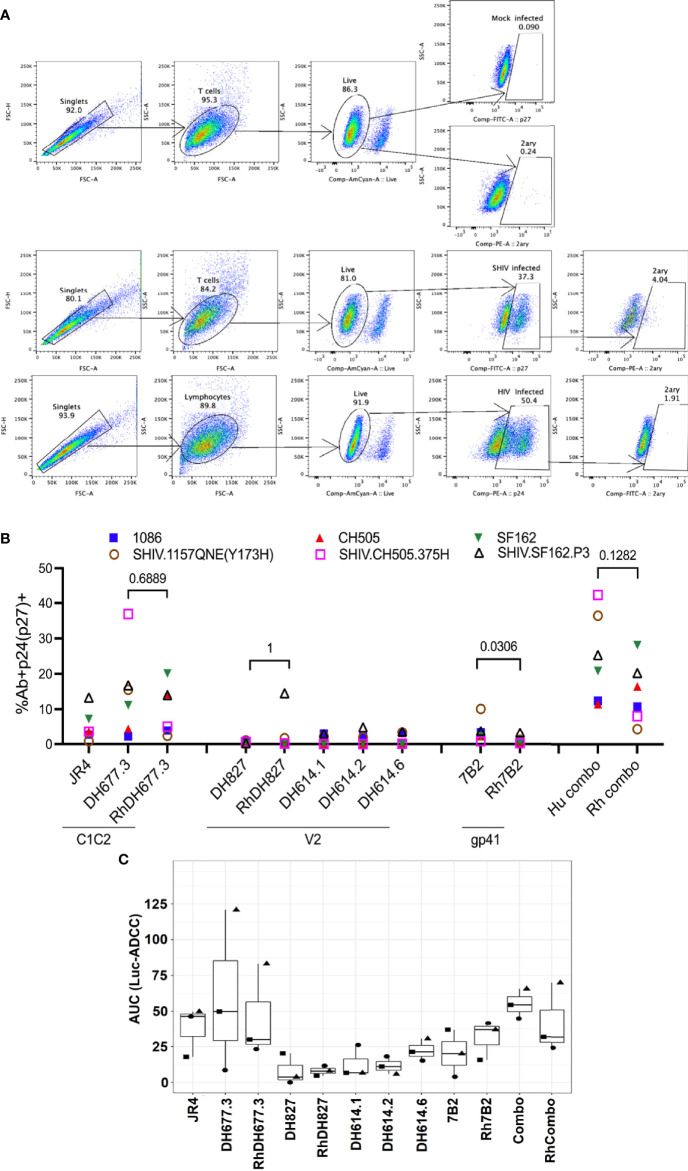
ADCC activities of rhesusized mAb variants. **(A)** The gating strategy for the binding of mAbs to HIV.IMC-infected CEM.NKR cells and SHIV-infected A66 cells. The binding of mAbs to mock-infected cells (top), SHIV-infected A66 cells (middle) and HIV-infected CEM.NKR cells and (bottom). Cells were first gated for singlets (FSC-H vs. FSC-A) and T cells (SSC-A vs. FSC-A). The T cells were further analyzed for their uptake of the Live/Dead Aqua stain to determine live versus dead cells. Live cells were analyzed for the intracellular expression of p24 or p27. Each well was stained either with anti-p24 (CEM.NKR cells that were infected with HIV) or anti-p27 antibody (A66 cells infected with SHIV). Infected cells, p24+ (infected with HIV.IMCs) or p27+ (infected with SHIVs), were analyzed for binding of the tested mAb by staining with a secondary mAb (2ary). The far-right panel indicate 2ary Ab only (these are the well that lack primary Ab of interest) which shows that in presence of secondary antibody alone we did not detect any binding to either mock or infected cells. Mock-infected cells were used to properly set the gate for the infected cell population and the 2ary mAb. FSC, forward scatter; SSC, side scatter. **(B)** The percentage of infected cells bound by the tested mAb. The antibodies listed on the x-axis are grouped by the epitope specificity: C1C2 (RM JR4, DH677.3), V2 (DH827, RM DH614.1, RM DH614.2, RM DH614.6), gp41 immunodominant (gp41), and the combination (DH677.3, DH827, 7B2). Each color represents a different HIV-1 Infectious Molecular Clone (IMC) or SHIV. Each Ab/virus combination was tested once using a single well. **(C)** ADCC activities are shown as area under the curve (AUC) for each mAb calculated from dilution curves (starting concentration 50 µg/mL with 1:5 serial dilutions) against HIV.IMC-infected cells determined by a Renilla Luciferase-based ADCC assay (Luc-ADCC) with PBMCs from an HIV-1-seronegative individual as effectors at an E:T ratio of 30:1. Each symbol represents a different IMC. Each experiment in panel C was performed once with two biological replicates. Wilcoxon rank sum test was used to assess statistical significance; p-values less than 0.05 were considered significant.

Structural and SPR analyses indicate that the rhesusized mAb variants preserve the antigen binding properties of their human counterparts, and SPR binding confirms that their binding affinities to the low affinity RM and human FcγRs are comparable to mAbs of RM origin. To see if these features translate into ‘proper’ functional activity, we assessed their binding to HIV-infected CEM.NKR.CCR5 cells and SHIV-infected A66 cells using the gating strategy demonstrated in **Figure 7A**. We observed similar percentages of HIV-infected cells bound by each pair of human and rhesusized mAb (%Ab+p24/p27+) to each HIV-1 Infectious Molecular Clone (IMC) and SHIV tested here (**Figure 7B**). The percentage of infected cells bound by the combination of three mAbs, DH677.3 (C1C2), DH827 (V2) and 7B2 (gp41) was also similar between human and macaque. The human version of DH677.3 demonstrated a difference in binding to SHIV.CH505.375H- and SHIV.1157QNE(Y173H)-infected cells as compared to its rhesus counterpart. A similar increase in binding was observed with the V2-targeting RhDH827 Ab as compared to its human version to SHIV.SF162.P3-infected cells. Overall, there was similar binding of rhesusized mAbs to HIV-infected cells as compared to their human counterparts.

The ADCC activity of the rhesusized variants to their human counterparts was also compared using HIV-infected CEM.NKR.CCR5 cells as targets in a luciferase-based ADCC assay (49). Peripheral blood mononuclear cells (PBMCs) isolated from an HIV-1-seronegative individual were used as effector cells and subtype B HIV-1 SF162-, subtype C HIV-1 CH505- or 1086-infected cells were used as targets in the presence of serial dilutions of mAbs. We observed similar area under the curve (AUC) values for human and rhesus mAb pairs against the three IMCs tested here (**Figure 7C** **).** The analysis of ADCC activity for mAbs in the ADCC-GTL assay (51) also revealed similar magnitudes in AUC values for each individual mAb pair and for macaque and human mAb combinations (**Figure S5**).

**Figure 8:**
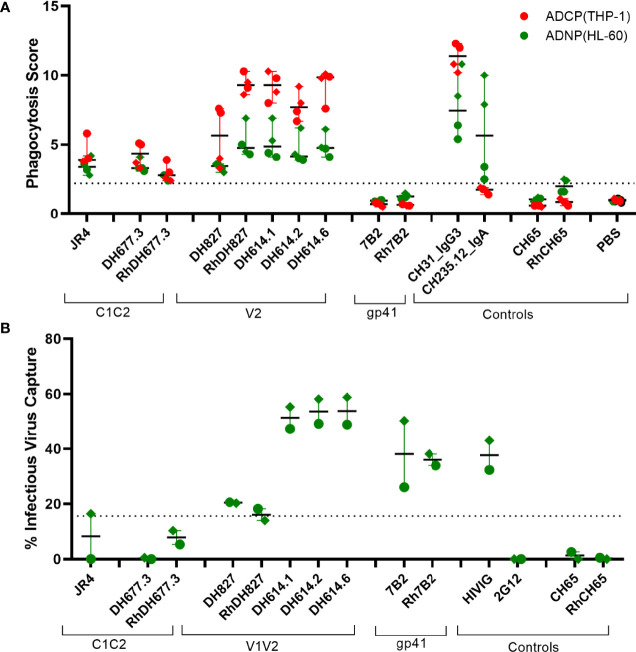
Virion binding and phagocytic activities of Rhesusized mAb **(A)** Antibody-dependent phagocytosis of monocytes using monocytic THP1 cell line and neutrophils using HL60 cell line with SHIV 1157(QNE)Y173H gp120-coated beads. **(B)** Binding of antibody variants to infectious SHIV1157(QNE)Y173H. Dotted lines represent positivity cutoffs of 15.6% and 2.2 for virus capture and ADCP/ADNP, respectively. The data represent an average of two independent experiments with two replicates for each experiment for ADCP/ADNP and one replicate for virion binding. Rhesus macaque antibody versions are marked with Rh. Positive controls (CH31 IgG3, CH235.12 IgA) and negative controls (7B2, Rh7B2, Ch65, RhCH65 and PBS) are shown.

The capacity of the C1-C2 and V2 specific rhesusized variants to mediate phagocytosis by monocytes (ADCP) and neutrophils (ADNP) was assessed using the THP-1 and HL-60 cell lines with SHIV 1157(QNE)Y173H gp120 protein coated microspheres, respectively (**Figure 8A**). Overall, the rhesusized Abs had similar levels of phagocytosis to their human counterparts. The V2 Abs had modestly higher phagocytosis scores compared to the C1C2 specific antibodies, likely due to improved epitope exposure on Env protein. To assess recognition of virus particles as one of the first steps in antibody effector function, we also assessed the capacity of all 3 antibody specificities to bind infectious SHIV 1157(QNE)Y173H virions (**Figure 8B**). As expected, the C1C2 antibody specificities did not bind and capture virus. However, the V1V2 and gp41 specific antibodies did capture infectious virions.

Taken together these data indicate that rhesusized mAb binding and ADCC to *in vitro* infected cells are comparable antibody Fc effector functions for human mAb counterparts as well as anti-HIV-1 antibodies of human or rhesus origin.

## Discussion

The number of antibodies and antibody-based therapeutics used clinically continues to grow (71, 72). Since many of these products are derived from antibodies from a nonhuman origin they need to be adapted to the human immune system, or ‘humanized’, before they can be used clinically. Simple grafting of an antibody’s complementary determining regions (CDRs) to a human antibody backbone sequence often leads to reduced antigen affinity either by omission of paratope residues outside of the CDRs or by subtle structural changes due to differences in the sequence of framework residues. Conversely, grafting of the entire heavy and light chain variable domain, V_H_ and V_L_, onto constant heavy and light chain backbones to make a chimera, while maintaining paratope structure introduces a greater number changes and potentially increases immunogenicity. This has typically made humanization of antibodies a multistep process in which the initial incarnation of a humanized antibody is subsequently modified to increase its affinity for antigen in order to match that of the parent mAb. The sequencing of the human genome has aided this process by making it possible to identify the closest germline sequence to minimize the number of needed residue changes and potentially decrease immunogenicity.

The close phylogenetic relationship between humans and nonhuman primates (NHPs), including Rhesus macaque (RM), *Macaca mulatta*, makes them an important animal model in the testing of new vaccines, antibodies or antibody based therapeutics. For human antibodies to be tested in RM this requires the reverse of humanization, i.e. rhesusization. The latter is essential to enable proper interaction with host immune system. Ideally this is done with the fewest number of sequence changes both to preserve the paratope structure of the parent mAb and to minimize immunogenicity. Here we have applied the rhesusization process to three human anti-HIV-1 antibodies recognizing Env which impact the virus predominantly by a Fc-effector mechanism (DH677.3, DH827, and 7B2) to facilitate their use in future NHP challenge studies.

We were able to obtain crystal structures of antigen complexes for two of our rhesusized variants in conditions similar to those used to obtain the structures of their human counterparts. This allowed us to perform a detailed analysis of the complex interface and to detect any changes introduced in the rhresusization process. Some slight differences were seen in the RhDH677.3 complex in contacts made by the Fab outside of the CDRs. Framework residues from the human mAb contributed slightly more to the interface than the corresponding residues from the rhesusized version. Interestingly, these differences could largely be attributed to positions that differed between the human and macaque germline sequences and are reflected in the total interface BSA, 1800 Å^2^ for RhDH677.3 and 1984 Å^2^ for DH677.3, and in the approximately 8-fold reduction in affinity for RhDH677.3 to antigen. Thus, for antibodies that involve framework residues in antigen engagement, rhesusization within framework areas should to be done carefully to avoid losses that can potentially lead to a decrease of affinity to antigen. Similar conclusions could be drawn from the comparison of the human and macaque versions of DH827 although a one residue difference in sequence in the light chain and different resolutions for the two structures made the comparison more difficult.

Importantly, the Fc-functionality of the rhesusized mAbs were fully preserved. SPR results confirmed that the rhesusized mAbs had affinities to both macaque and human FcγRs similar to mAbs originally isolated from RM. Rhesusized mAbs also bound to HIV-1 infected cells at levels comparable to those of their human counterpart. They also displayed ADCC, ADCP and ADNP activities in the same range as macaque mAbs of similar specificity.

In conclusion, reducing immunogenicity potentially comes at the cost of reduced affinity to antigen. Even highly somatically mutated species matched antibodies can elicit immune reactions that remove them from circulation. This has been the case in macaques with the introduction of anti-SIV mAbs (73) and in humans with the introduction of broadly neutralizing antibodies against HIV-1, PG9 in clinicaltrials.gov NCT01937455 (74) and VRC07 in VRC 603, clinicaltrials.gov NCT03374202 (75). Rhesusization can potentially minimize the impact of such off-target reactions and extend their half-life in sera when they are evaluated in NHP models (33), but anti-idiotype immune responses are still possible (76). Rhesusization also places the antibody specificity within the context of the host immune response affording a more direct comparison to vaccine elicited antibodies of similar specificity. This may enable use of the RM model to more accurately test antibody correlates of protection from HIV-1 infection identified in human vaccine trials and more generally antibodies and antibody based therapeutics that utilize Fc mediated effector functions as part of their mechanism of action.

## Data Availability Statement

The datasets generated and/or analyzed during the current study are available in the Protein Data Bank (PDB) http://www.rcsb.org under admission codes: 6OZ2 and 6OZ4.

## Author Contributions

WT, DN, GT, GF, and MP designed, performed research, and analyzed the data, MT, SJ, and GF designed, performed, and analyzed cell binding and ADCC data. AC, YC, and MA designed, performed, and analyzed SPR binding data. KW helped with rhesus germline gene inference. GT, KS, and JP designed and produced RM IgG variants. DG and GT designed and analyzed phagocytosis experiments. AD, GL, DE, MM, VB, and SM helped design the mAb selections and experiments. JT and AP provided the SHIV QNE and helped with experimental design. WT, DN, and MP wrote the manuscript and all authors provided comments or revisions. All authors contributed to the article and approved the submitted version.

## Funding

Funding for this study was provided by the National Institute of Health grants: P01 AI120756 to GT, R01 AI116274 to MP, R01 AI129769 to MP, with support from the Duke Center for AIDS Research P30 AI064518. The funders had no role in study design, data collection and analysis, decision to publish, or preparation of the manuscript and the contents of this publication are solely the responsibility of the authors.

## Author Disclaimer

The views expressed in this presentation are those of the authors and do not reflect the official policy or position of the Uniformed Services University, US Army, the Department of Defense, or the US Government.

## Conflict of Interest

The authors declare that the research was conducted in the absence of any commercial or financial relationships that could be construed as a potential conflict of interest.

## Publisher’s Note

All claims expressed in this article are solely those of the authors and do not necessarily represent those of their affiliated organizations, or those of the publisher, the editors and the reviewers. Any product that may be evaluated in this article, or claim that may be made by its manufacturer, is not guaranteed or endorsed by the publisher.
