# Supplementary material for: Structure and Fc-Effector Function of Rhesusized Variants of Human Anti-HIV-1 IgG1s
Source: Front Immunol. 2022 Jan 6;12:787603. doi: 10.3389/fimmu.2021.787603 (PMC8770954; doi:10.3389/fimmu.2021.787603)
Supplement: Supplementary file 1 [file DataSheet_1.pdf]

Supplementary Material

Supplementary Material

1 Supplementary Figures and Tables

1.1 Supplementary Tables

Table S1. Equilibrium binding constants for IgG1 variants to RM and human FcγRs

	Rhesus macaque K_D equil (μM)					
	FcγRIIa-1	FcγRIIa-2	FcγRIIa-4	FcγRIIb-1	FcγRIIIa-1(I^{158})	FcγRIIIa-3(V^{158})
RhDH677.3	4.7	4.9	6.2	3.7	1.1	1.5
Rh7B2	6.0	5.5	7.7	3.7	1.0	1.6
RhDH827	5.7	5.5	6.9	3.7	1.3	1.9
RM JR4	4.9	4.5	6.9	3.1	1.1	1.6
RM DH614.1	5.0	4.7	6.8	3.0	0.8	1.3
RM DH614.2	4.9	4.7	7.0	3.1	0.9	1.3
RM DH614.3	4.4	4.2	6.5	2.8	0.9	1.4

	Human K_D equil (μM)				
	FcγRIIa(^{131})	FcγRIIa(H^{131})	FcγRIIb	FcγRIIIa(F^{158})	FcγRIIIa(V^{158})
RhDH677.3	4.6	1.5	7.9	3.8	0.8
Rh7B2	2.9	4.4	8.5	3.8	1.8
RhDH827	4.6	3.7	7.2	4.1	2.4
RM JR4	2.0	3.6	8.3	3.9	1.7
RM DH614.1	2.7	3.5	6.6	3.9	1.6
RM DH614.2	1.9	4.5	10.1	3.9	1.6
RM DH614.3	1.7	3.9	6.9	3.9	1.4

Table S2. Details of the RhDH677.3, the DH677.3, the RhDH827 and the DH827 interfaces based on the RhDH677.3 Fab-gp120$_{93TH057}$core$_e$-M48U1, the DH677.3 Fab-gp120$_{93TH057}$core$_e$-M48U1, the RhDH827 Fab-V2 peptide and the DH827 Fab-V2 peptide complex structures as calculated by the EBI PISA server (http://www.ebi.ac.uk/msd-srv/prot_int/cgi-bin/piserver). The values for the two copies in the asymmetric unit of the DH677.3 complex are averaged in the table while those of the first copy only (with M48U1 bound) are shown for the RhDH677.3 complex.

		RhDH677.3Fab -gp120$_{93TH057}$ core$_e$-M48U1	DH677.3 Fab - gp120$_{93TH057}$ core$_e$-M48U1	RhDH827 Fab - V2peptide	DH827 Fab - V2peptide
**Buried Surface Area, Å^2**	**gp120 total**	**876**	**934**	**852**	**817**
	7/8-stranded β sheet	226	261	0	0
	Layer 1	530	540	0	0
	Layer 2	120	133	0	0
	Layer 3	0	0	0	0
	V2 loop	0	0	852	817
	**Heavy chain total**	**463**	**477**	**492**	**516**
	FWR	0	0	0	0
	CDR H1	10	18	51	53
	CDR H2	96	81	184	195
	CDR H3	357	378	257	268
	**Light chain total**	**461**	**483**	**293**	**307**
	FWR	59	90	2	1
	CDR L1	246	229	124	138
	CDR L2	28	28	37	35
	CDR L3	128	136	130	133
	**Heavy and light chain total**	**924**	**960**	**785**	**823**

1.2 Supplementary Figures

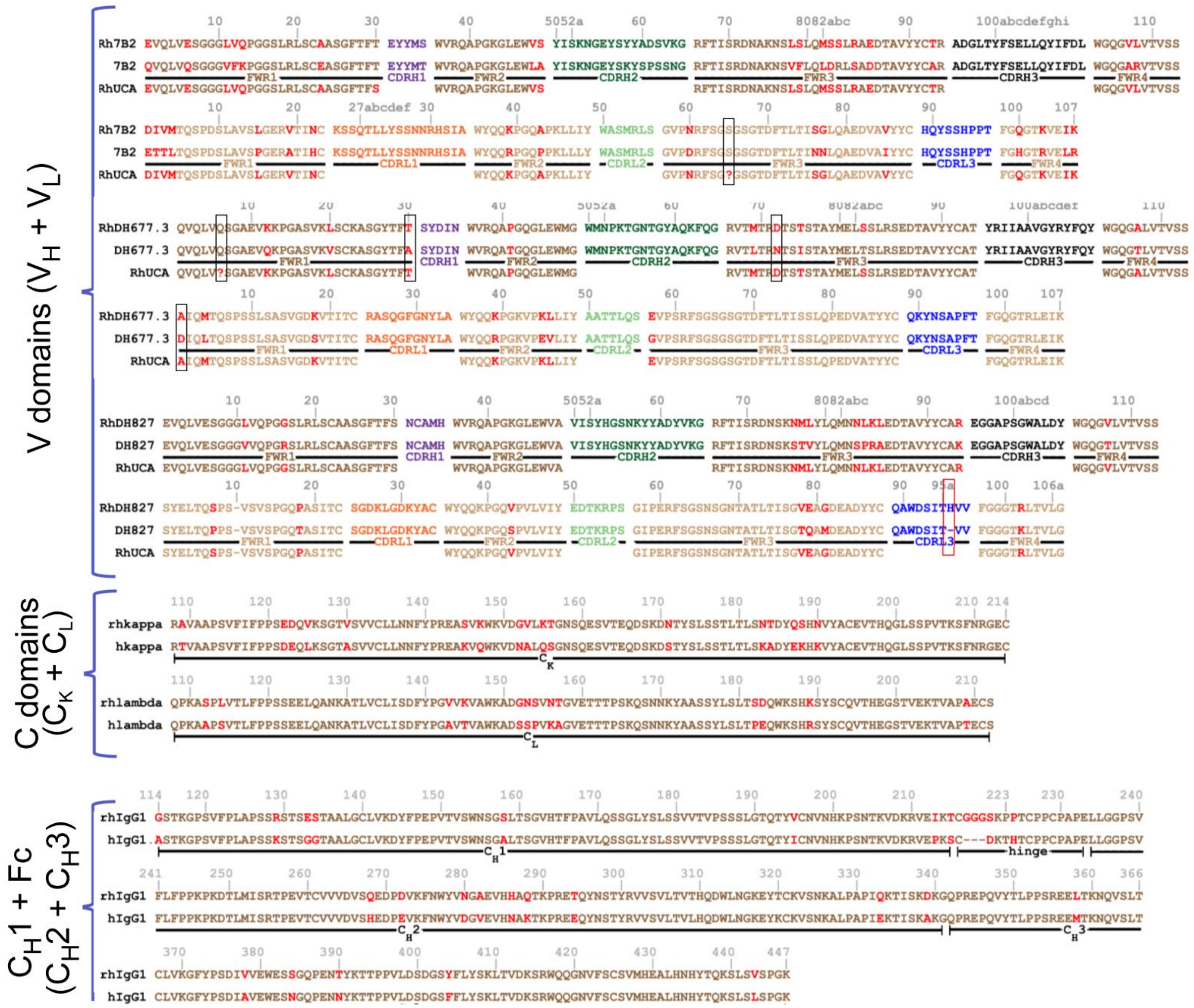

Figure S1. Sequences for rhesusized variants of Rh7B2, RhDH677.3 and RhDH827. Variable domains of the three rhesusized mAbs with rhesusized sequence, human sequence and rhesus unmutated common ancestor sequence (RhUCA) for each antibody (top). Constant domains for the lambda and kappa light chains as compared to the human sequences (middle). Constant domains for the heavy chain (C_H1, C_H2, and C_H3) as compared to the human sequence. Residues that are different between macaque and human are highlighted in red. CDRs are colored and labelled as shown.

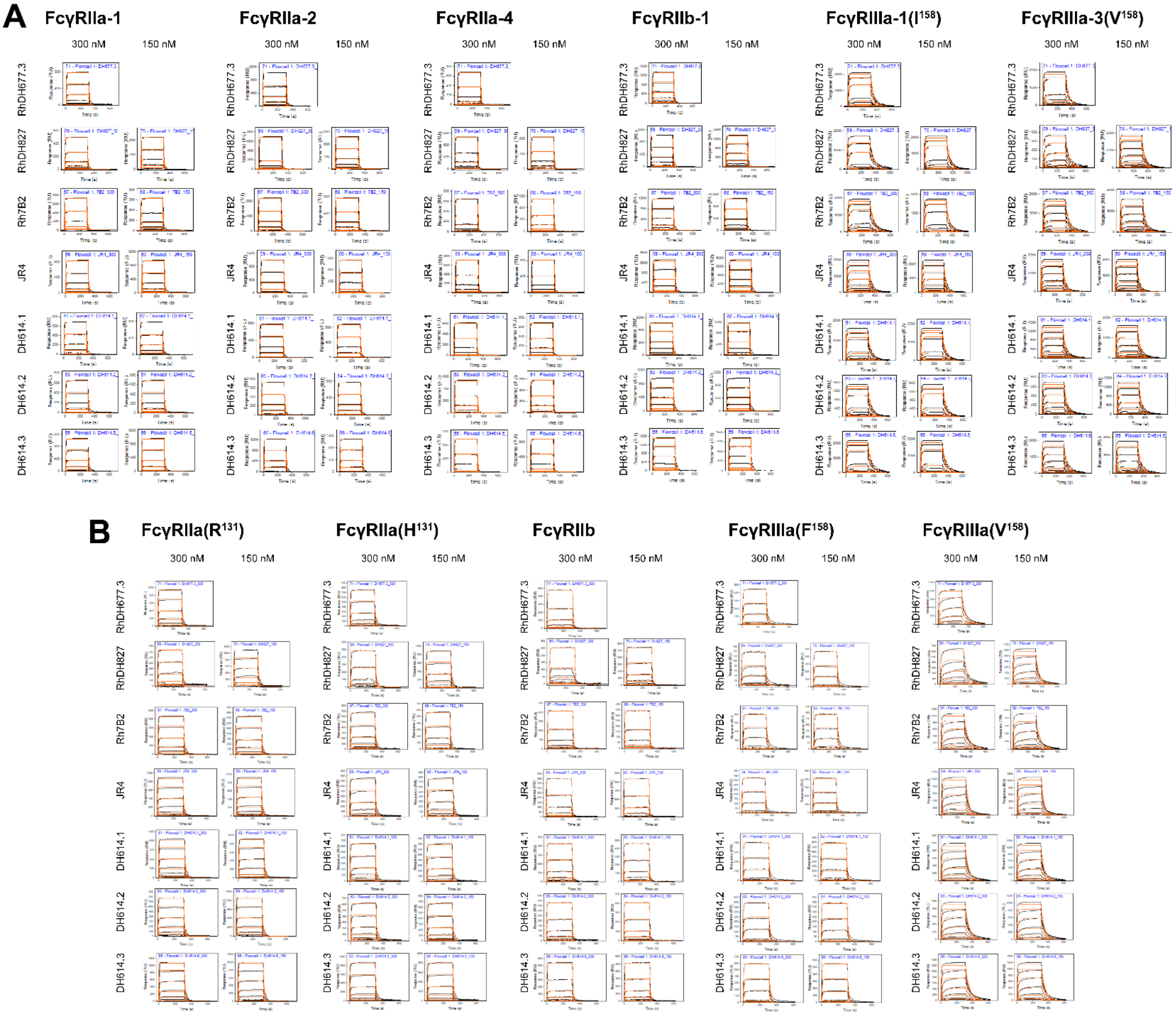

Figure S2. BLI sensorgrams (black) and curve fits (red) of binding of IgG1 variants to RM and human FcγRs. Antibodies were printed at two concentrations (300 and 150 nM), shown in side by side columns.

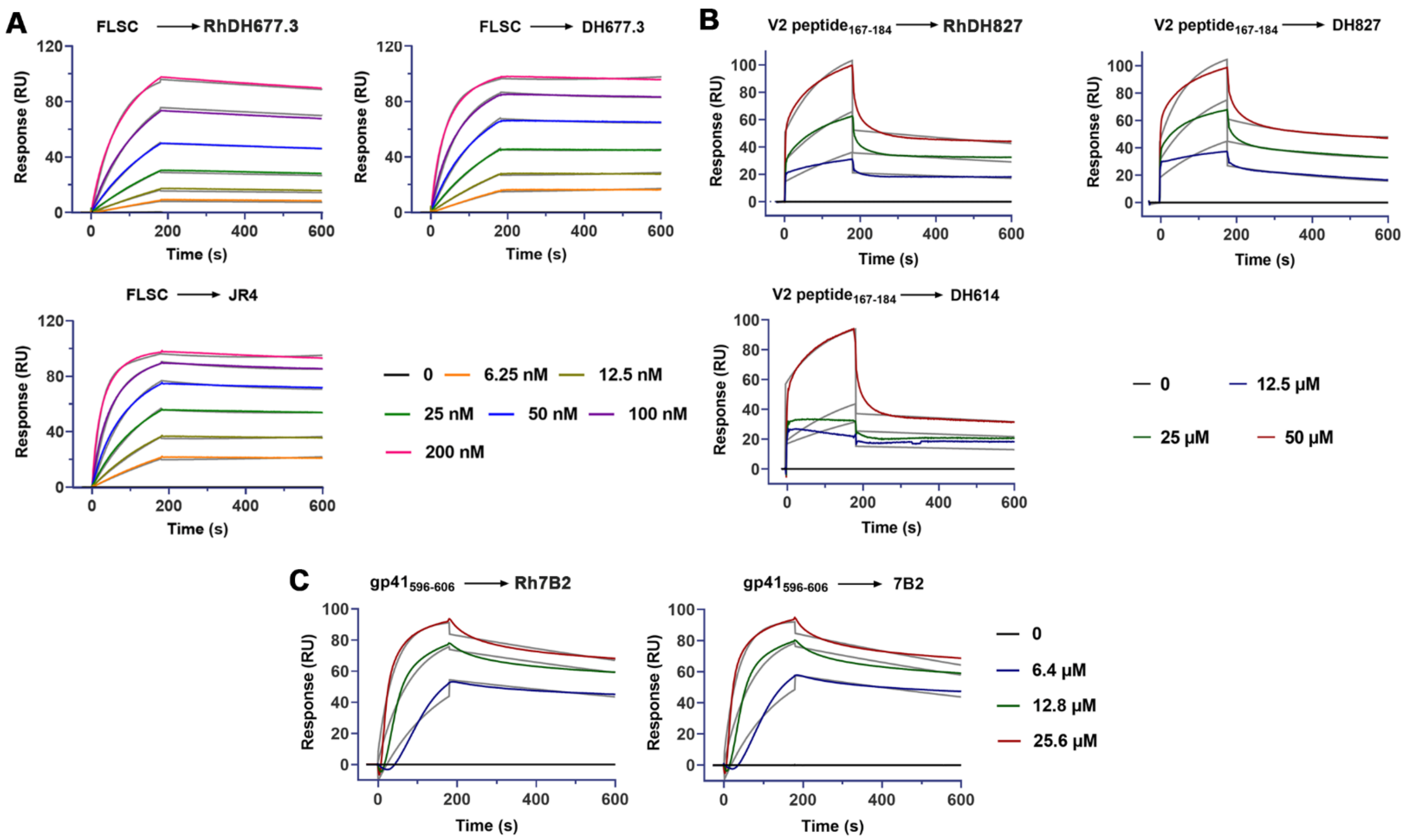

Figure S3. SPR binding curves (black) and curve fits (color) for the binding of IgG1 variants to HIV-1 Env antigens.

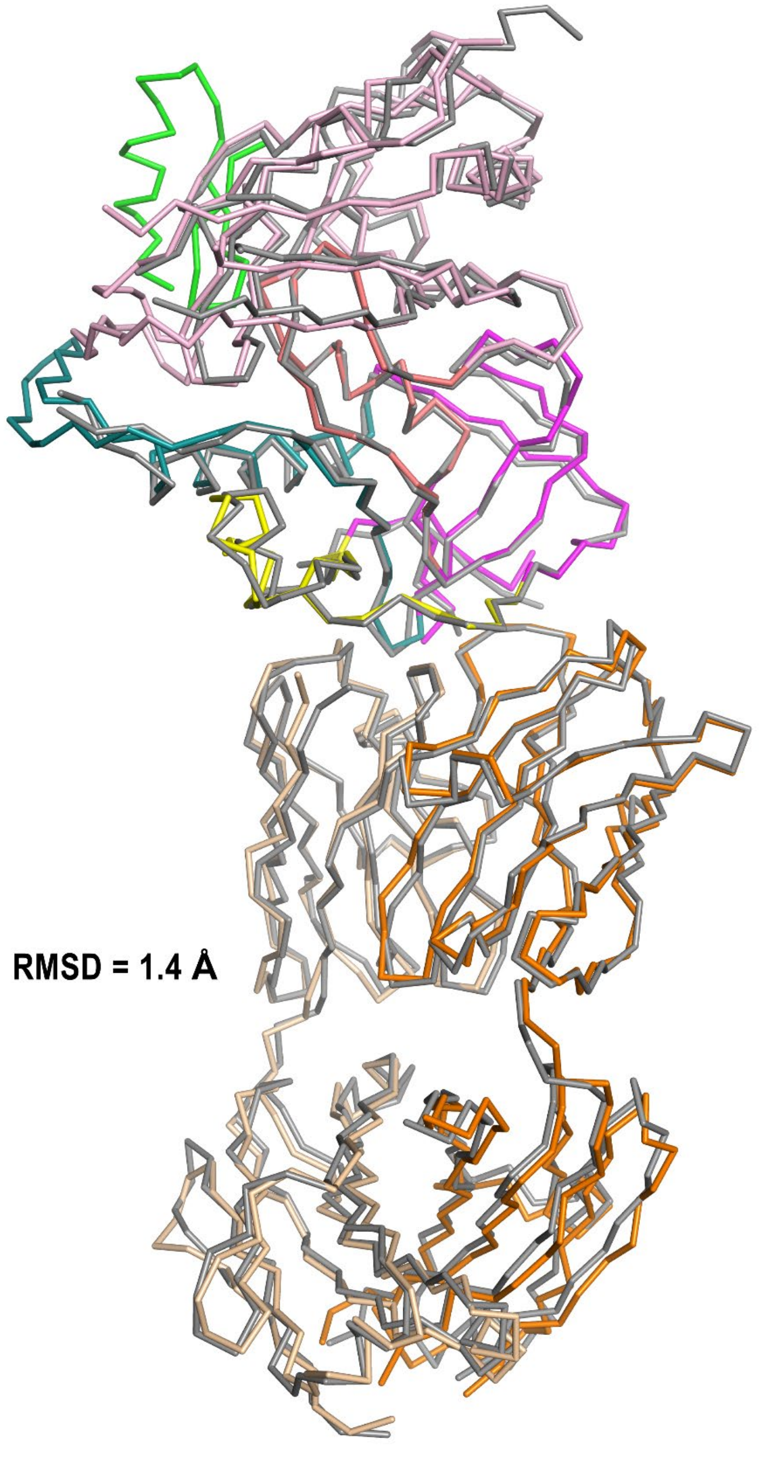

Figure S4. Comparison of the two copies of the RhDH677.3 Fab-gp120$_{93TH057}$ core$_e$-M48U1 complex. Of the two copies present in asymmetric unit of the RhDH677.3 Fab-gp120$_{93TH057}$ core$_e$-M48U1 complex only one had the CD4 mimetic M48U1 bound (complex 1). Despite this difference the root mean square deviation (RMSD) between the complex copies (RhDH677.3 Fab-gp120$_{93TH057}$ core$_e$) is 1.4 Å for main chain residues of the full complex and 1.1 Å for the main chain residues of the variable part of the Fab and gp120 in the complex. In both copies the β-sandwich shows good rigidity and was found largely independent of CD4 bound status. In contrast mobile layers 1 and 2 have more flexible conformations that can vary with the presence or absence of CD4 or

gp41. The two complexes in the asymmetric unit of the RhDH677.3, complex 1 with the CD4 mimetic M48U1 bound and complex 2 with no CD4 mimetic bound, had almost identical heavy chain buried surface areas (BSAs) (complex 1, BSA of 404 Å^2 and complex 2, BSA of 401 Å^2. **Table S1**). But there were differences in how the two complexes arrived at these totals with complex 1 having slightly larger contributions from gp120 inner domain layers 1 and 2, 216 and 14 Å^2 versus 202 and 11 Å^2 respectively, and complex 2 having a higher contribution from the 7-stranded β-sandwich, 187 versus 174 Å^2. This difference in binding was more apparent in the light chain with complex 1 having a layer 1 BSA of 314 Å^2 and a layer 2 BSA of 105 Å^2 while complex 2 had a layer 1 BSA of 277 Å^2 and a layer 2 BSA of 121 Å^2; the light chain 7-stranded β-sandwich BSAs were largely identical, 52 and 49 Å^2 respectively. The total RhDH677.3 gp120 BSA was lower for complex 2, 848 Å^2 versus 876 Å^2, even though contact residues were identical in both structures (layer 1 residues 53, 71-80 and 82, layer 2 residues 219-222, and 7-stranded β-sandwich residues 84, 223-224, 244-246, and 491-492). RhDH677.3's greater use of the 7-stranded β-sandwich in binding to gp120 in complex 2 may therefore be a consequence of conformational changes within layers 1 and 2 with and without M48U1 bound.

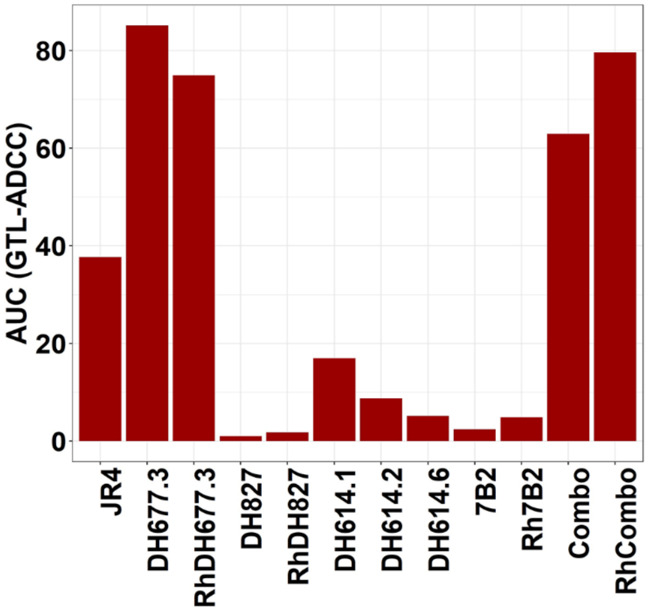

Figure S5. ADCC activities against gp120-coated cells obtained using a GTL-ADCC assay. ADCC activities are shown as area under the curve (AUC) for each mAb calculated from dilution curves (starting concentration 50 µg/mL with 1:5 serial dilutions) against gp120-coated cells with PBMCs from an HIV-1-seronegative individual as effectors at an E:T ratio of 30:1. Each bar represents a different antibody. The experiment was performed once with two biological replicates.
